# Design, Synthesis and Evaluation of Fused Bicyclo[2.2.2]octene as a Potential Core Scaffold for the Non-Covalent Inhibitors of SARS-CoV-2 3CL^pro^ Main Protease

**DOI:** 10.3390/ph15050539

**Published:** 2022-04-27

**Authors:** Barbara Herlah, Andrej Hoivik, Luka Jamšek, Katja Valjavec, Norio Yamamoto, Tyuji Hoshino, Krištof Kranjc, Andrej Perdih

**Affiliations:** 1 National Institute of Chemistry, Hajdrihova ulica 19, SI-1000 Ljubljana, Slovenia; barbara.herlah@ki.si (B.H.); katja.valjavec@ki.si (K.V.); 2 Faculty of Pharmacy, University of Ljubljana, Aškerčeva 7, SI-1000 Ljubljana, Slovenia; 3 Faculty of Chemistry and Chemical Technology, University of Ljubljana, Večna pot 113, SI-1000 Ljubljana, Slovenia; andrej.hoivik@gmail.com (A.H.); luka.jamssek@gmail.com (L.J.); 4 Department of Microbiology, Tokai University School of Medicine, 143 Shimokasuya, Isehara 259-1193, Kanagawa, Japan; n-yamamoto@tsc.u-tokai.ac.jp; 5 Graduate School of Pharmaceutical Sciences, Chiba University, 1-8-1 Inohana, Chuo-ku, Chiba 260-8675, Chiba, Japan; hoshino@chiba-u.jp

**Keywords:** bicyclo[2.2.2]octenes, molecular scaffolds, SARS-CoV-2, 3CL^pro^ main protease, COVID-19 antiviral drugs

## Abstract

The emergence of SARS-CoV-2, responsible for the global COVID-19 pandemic, requires the rapid development of novel antiviral drugs that would contribute to an effective treatment alongside vaccines. Drug repurposing and development of new molecules targeting numerous viral targets have already led to promising drug candidates. To this end, versatile molecular scaffolds with high functionalization capabilities play a key role. Starting with the clinically used conformationally flexible HIV-1 protease inhibitors that inhibit replication of SARS-CoV-2 and bind major protease 3CL^pro^, we designed and synthesized a series of rigid bicyclo[2.2.2]octenes fused to *N*-substituted succinimides to test whether this core scaffold could support the development of non-covalent 3CL^pro^ inhibitors. Inhibition assays confirmed that some compounds can inhibit the SARS-CoV-2 main protease; the most promising compound **11a** inhibited 3CL^pro^ in micromolar range (IC_50_ = 102.2 μM). Molecular simulations of the target-ligand complex in conjunction with dynophore analyses and endpoint free energy calculations provide additional insight and first recommendations for future optimization. The fused bicyclo[2.2.2]octenes can be used as a new potential starting point in the development of non-covalent SARS-CoV-2 3CL^pro^ protease inhibitors and the study also substantiates the potential of this versatile scaffold for the development of biologically active molecules.

## 1. Introduction

In 2020 and 2021, severe acute respiratory syndrome coronavirus 2 (SARS-CoV-2) has become one of the leading pulmonary diseases and has greatly affected the lives of virtually the whole human population [1,2]. As of early 2022, the virus was responsible for more than 6 million deaths.

SARS-CoV-2 genetically closely resembles the severe acute respiratory syndrome coronavirus (SARS-CoV), which was responsible for a much smaller-scale epidemic in 2003, limited predominantly to the Asian continent [3]. SARS-CoV and SARS-CoV-2, as well as the Middle East respiratory syndrome coronavirus (MERS-CoV), all belong to the family of *Coronaviridae*. All three of them, being zoonotic viruses, have the ability to cause a severe infection in humans. In contrast, human CoVs HCoV-NL63, HCoV-229E, HCoV-OC43, and HCoVHKU1 are mostly responsible only for milder pulmonary infections [4]. As of right now, the most indicative symptoms of COVID-19 infection in patients are high fever, cough, loss of taste and smell, and uncontrolled respiratory sickness that often requires intensive care [5].

Coronaviruses are single-stranded positive-sense RNA viruses and can be categorized into four species: alpha, beta, gamma, and delta. The most recent SARS-CoV-2 belongs to the beta species and is identified to affect humans [6]. Its genome encodes two overlapping polyproteins—pp1a (replicase 1a, 450 kDa) and pp1ab (replicase 1ab, 750 kDa)—that are required for the viral replication and transcription [7,8]. The functional polypeptides are released from the polyproteins by extensive proteolytic processing, predominately carried out by the 33.8 kDa main proteinase (M^pro^), also termed 3C-like proteinase (3CL^pro^). The 3CL^pro^ cleaves the protein at 11 (or more) conserved sites involving Leu, Gln ↓ (Ser, Ala, Gly) sequences (↓ indicated the cleavage site), starting with the enzyme’s own catalytic cleavage from pp1a and pp1ab [8,9]. The importance of 3CL^pro^ for SARS-CoV-2 viral function and replication, as well as the lack of a very similar homologous gene in humans, make the protease a promising target in antiviral drug development and design [10].

After the wide spread of the pandemic in mid-2020, science has made great advances to combat the virus. Late 2020 saw the launch of several efficient vaccines, which successfully prevented countless deaths. Concurrently, the race to develop the first antivirals is also taking place, as there is a strong need for efficient anti-COVID-19 drugs to treat unvaccinated patients with severe symptoms, as well as infected vaccinated patients with weaker immune response to vaccines, or immunocompromised patients. Such therapeutic intervention would reduce the hospital burden caused by the COVID-19 patients [3,8].

Even intensive past efforts to develop drugs against HIV cannot compare with the scale of drug design now conducted against SARS-CoV-2 [4]. One of the main strategies utilized, especially in the development of small molecules, is the repurposing of existing antivirals, as they have already been tested and approved for their nontoxicity. This also somewhat increases the likelihood of passing the preclinical and clinical stages of drug design. In November 2021, molnupiravir, the first drug for the treatment of COVID-19 was approved in the UK [11]. It acts as a prodrug which, upon activation in the body, inhibits the viral reproduction by being falsely inserted into the newly forming RNA molecule synthesized by the RNA-directed RNA polymerase, thus preventing the further replication of the virus’ genetic material. The molecule was discovered by repurposing potential antiviral drug targeting Venezuelan equine encephalitis virus (VEEV) as well as influenza virus.

In principle, to search for therapeutic options against the SARS-CoV-2, all CoV enzymes and proteins involved in the viral replication and control of the host cellular mechanisms are potential targets [4]. Two large polyproteins mentioned previously, pp1a and pp1ab, are cleaved and transformed into mature non-structural proteins (nsps) by the two proteases 3C-like protease or main protease (3CL^pro^) and papain-like protease (PL^pro^) encoded by the open reading frame (ORF) 1a/b [12]. Both proteins are crucial for the viral replication and control of the host cell responses and are, therefore, important targets for antiviral drug development. The sequences of 3CL^pro^ in SARS-CoV and SARSCoV-2 are 96% identical, and the minimal differences between the two enzymes appear on the surface of the proteins. Therefore, inhibitors against SARS-CoV 3CL^pro^ are expected to also inhibit SARS-CoV-2 3CL^pro^. Applying this information has led to the development of SARS-CoV-2 3CL^pro^ covalent inhibitors PF-07304814 (lufotrelvir) and PF-07321332 (studied together with ritonavir), which both showed favorable results in human clinical trials.

In recent years, numerous small molecules, peptides, and peptidomimetics have been developed that are able to inhibit SARS-CoV or both SARS-CoV and MERS-CoV 3CL^pro^, or even both proteases 3CL^pro^ and PL^pro^ [4]. SARS-CoV-2 PL^pro^ has an 83% sequence identity with SARS-CoV PL^pro^. Although this is not as high as 3CL^pro^, most of the different residues are located on the surface. Therefore, it is very likely that the SARS-CoV PL^pro^ inhibitors could also be active against PL^pro^ of SARS-CoV-2 [4]. PL^pro^ is another important target that inhibits not only viral replication, but also the dysregulation of the signaling cascades in infected cells that can lead to cell death of the neighboring uninfected cells [13].

Investigation of two approved drugs for the treatment of HIV infections, namely HIV-1 protease inhibitors lopinavir and ritonavir, displayed their potential to act as SARS-CoV 3CL^pro^ inhibitors [14]. Further results demonstrated that nelfinavir, another clinically used HIV-1 protease inhibitor, was also able to inhibit replication of SARS-CoV-2 in a dose-dependent manner as a noncovalent inhibitor]. Nelfinavir was predicted to bind SARS-CoV-2 3CL^pro^, and nelfinavir in combination with cepharanthine significantly reduced the viral RNA levels [15]. Further results on a wide range of clinically used HIV-1 protease inhibitors indicated this class can inhibit the replication of SARS-CoV-2 and can target its main protease [14,16,17,18,19,20,21]. These data prompted us to search for new classes of molecules that would target the SARS-CoV-2 main protease via noncovalent inhibition. Adaptable molecular scaffolds with high functionalization capabilities play a key role in medicinal chemistry research, especially during the compound optimization process. They have to allow the correct positioning of key interactions for the productive molecular recognition process between ligand and target; on the other hand, they should not exhibit significant conformational flexibility of their core structure.

**Figure 1:**
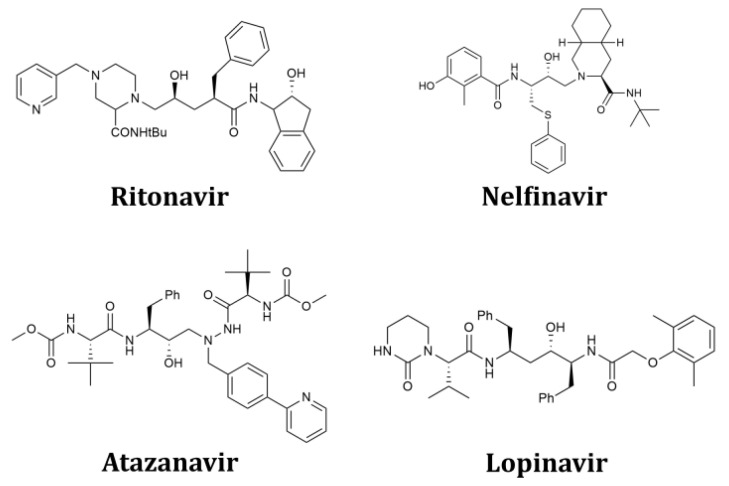
Structures of the clinically used HIV-1 inhibitors that were predicted to bind the 3CL^pro^ main protease of SARS-CoV-2 and formed the design starting point.

In this study, we report our efforts to expand the known chemical space of scaffolds that could target the SARS-CoV-2 3CL^pro^ as non-covalent substrate inhibitors. The HIV-1 protease inhibitors in Figure 1 served as a starting point for the design and synthesis of a series of compounds containing a rigid ethenopyrrolo[3,4-*f*]isoindole scaffold (i.e., bicyclo[2.2.2]octene fused to two *N*-substituted succinimide moieties). Subsequently, its potential as a core scaffold for the development of non-covalent SARS-CoV-2 3CL^pro^ inhibitors was evaluated in an inhibition assay. To gain deeper insight into protein–ligand molecular recognition of proteins and ligands, we also performed a molecular simulation of the most promising inhibitor identified. In addition, the study also aims to further highlight the utility of this scaffold in the development of biologically active molecules.

## 2. Results and Discussion

### 2.1. Structure-Based Design of Fused Bicyclo[2.2.2]octene as a Core Scaffold of SARS-CoV-2 3CL^pro^ Inhibitors

In our efforts to design novel compounds which could non-covalently bind to SARS-CoV-2 3CL^pro^ main protease, we first scanned its active site using the apo site grid functionality available in LigandScout [22]. For this purpose, various molecular probes corresponding to different pharmacophore features (i.e., hydrogen bond acceptor, hydrogen bond donor, positive ionizable, negative ionizable, hydrophobic probes, etc.) scanned SARS-CoV-2 3CL^pro^ active site and generated contours of the corresponding molecular interaction fields (MIFs) to pinpoint favorable interacting areas. This provided the first indication of the type of interactions and their positions in the active site that would lead to productive binding of potential 3CL^pro^ inhibitors. The generated MIFs showed that the active site of 3CL^pro^ provides many opportunities for hydrophobic interactions and has several hydrogen bond acceptor regions. On the other hand, favorable hydrogen bond donor regions were less present (Figure S1).

**Figure 2:**
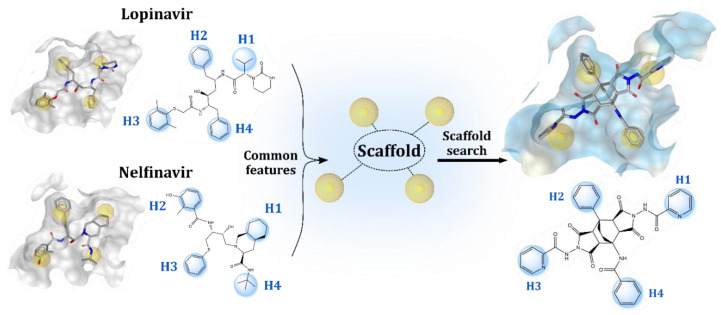
Structure-based design of fused bicyclo[2.2.2]octene as to SARS-CoV-2 3CL^pro^ inhibitors. HIV-1 protease inhibitors nelfinavir and lopinavir contain four hydrophobic moieties that, according to docking study, can fit into sub-pockets of the 3CL^pro^ active site. Model compound **11a** with fused bicyclo[2.2.2]octene scaffold is able to mimic this pharmacophore pattern when docked in the 3CL^pro^ active site.

Repurposing of already marketed drugs has become a valuable means of obtaining drugs for new therapeutic areas with already evaluated toxicology. This can increase the chances of such molecules passing through the preclinical and clinical stages of drug development [23]. Previous studies have shown that marketed drugs targeting HIV-1 protease have the potential to inhibit SARS-CoV-2 3CL^pro^ [16,17,18,20,21]. Structurally, all these compounds contain three to four hydrophobic moieties (labelled H1–H4) that would allow the formation of hydrophobic interactions with the 3CL^pro^ binding site sub-pockets. These moieties are shown in Figure 2 for nelfinavir and lopinavir.

We began our molecular design by docking the HIV-1 protease inhibitors shown in Figure 1 [24] in the active site of the crystal structure of SARS-CoV-2 3CL^pro^ using the GOLD docking software [8]. We then examined the proposed binding positions of each compound and discovered that each docked similarly, as shown for nelfinavir and lopinavir (Figure S2). In addition, we noticed that the H1–H4 regions of all these molecules interacted with the same hydrophobic amino acids of the active site 3CL^pro^ sub-pockets, consisting mainly of residues Thr25, Met49, Ala142, Met165, Gln189, and Ala191. This allowed us to outline an initial pharmacophoric requirement, i.e., that such placement of hydrophobic regions/interactions could be considered a favorable feature and we, therefore, wanted to incorporate it into the design of new compounds (Figure 2).

Most HIV-1 protease inhibitors, including those shown in Figure 1, can be classified as peptidomimetics that have a flexible scaffold allowing some freedom in positioning the H1–H4 components in space. Due to the considerable conformational space of such molecules, their binding is often less favorable because of the entropic penalty [25]. Moreover, the peptide-like structures often suffer from low bioavailability and possibly immunogenicity [25,26]. Therefore, in drug development, it is often recommended to use a rigid core scaffold with minimal flexibility [27]. In search of such a scaffold, we considered the bicyclo[2.2.2]octene skeleton as a potential core candidate that is readily amenable to various derivatizations and, in addition to its rigidity, allows for the incorporation of proposed pharmacophore requirements, thereby optimizing interactions with the hydrophobic components of the active site of 3CL^pro^.

Our previous extensive experience with bicyclo[2.2.2]octene skeletons [28,29,30] has shown that they can provide a very robust and chemically inert nonpeptide skeleton appropriate for further derivatizations. Bicyclo[2.2.2]octenes with fused succinic anhydride rings (i.e., ethenopyrrolo[3,4-*f*]isoindoles) can be thus used as starting compounds for transformations with variously substituted primary amines or hydrazine and its derivatives (having one NH_2_ group unsubstituted); additionally, they are nowadays quite common fragments of various polymeric materials [31] and zeolites [32,33,34,35]. In the final step of the molecular design, we combined the pharmacophoric requirements of the H1–H4 units with the proposed rigid bicyclo[2.2.2]octene scaffold fused to two succinimide rings to form a model compound **11a**, which we were also able to synthesize. We then docked the model compound to the active site of SARS-CoV-2 3CL^pro^ using the same settings as for nelfinavir and lopinavir. Visualization showed uniform docking positions with hydrophobic H1–H4 moieties occupying the same regions as nelfinavir and lopinavir (Figure 1) and forming hydrophobic interactions with residues, such as Thr25, Met49, Ala142, Met165, and Ala191 (Figure S3, right). Compound **11a** also completely occupied the place of the covalently bound inhibitor in the 6LU7 crystal structure (Figure S4). We decided to synthesize the model compound **11a** along with a series of its analogs **11b**–**o** (Table 1) to further extend the SAR data.

### 2.2. Synthesis of Fused Bicyclo[2.2.2]octenes

Only three years after the initial discovery of the [4+2] cycloaddition reactions by Diels and Alder in 1928 [36], their scope was extended by the same authors to the transformation of 2*H*-pyran-2-ones with maleic anhydride, yielding so-called double cycloadducts [37]. The synthetic applicability of this transformation was then, however, overlooked for many decades, and only in the 1980s it re-emerged in the literature. Variously substituted bicyclo[2.2.2]octenes were thus prepared from corresponding 2*H*-pyran-2-one derivatives and maleic anhydride [32,33,34,35,38]. There are also some alternative synthetic strategies towards bicyclo[2.2.2]octenes, such as reactions of maleic anhydride with thiophene-1,1-dioxide (with the elimination of SO_2_) [39,40] or with a germanium analogue [41]. Other pathways include reactions of maleic anhydride with substituted 3a,7a-dihydro-1,3-isobenzofurandione [42], with 4-hydroxy-3,4-diphenylcyclopent-2-en-1-one [43], with (*E*)-(4-bromopenta-2,4-dien-2-yl)benzene [44], or with 2,4-dimethylcrotonaldehyde [45].

**Scheme 1:**
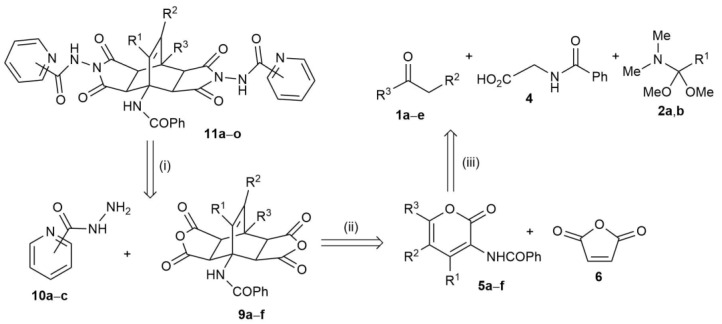
Retrosynthetic analysis of the required bicyclo[2.2.2]octenes **11a**–**o**.

Due to our extensive research in the field of the preparation of variously substituted bicyclo[2.2.2]octenes via Diels–Alder reactions of 2*H*-pyran-2-ones [28,29,30], we based the synthesis of the required derivatives **11** on a [4+2] cycloaddition of maleic anhydride with substituted 2*H*-pyran-2-ones. According to the retrosynthetic analysis (Scheme 1), we envisaged the following strategy to access the required adducts **11a**–**o**: (i) the first retrosynthetic step includes the application of three regioisomeric pyridylcarbohydrazides **10a**–**c** to derivatize the appropriate bicyclo[2.2.2]octenes **9a**–**f**; (ii) these are, in turn, formed in the second retrosynthetic step by a double one-pot Diels–Alder cycloaddition between maleic anhydride (**6**) and 3-benzoylamino-2*H*-pyran-2-one derivatives **5a**–**f**; (iii) **5** are obtained in the last retrosynthetic step from an appropriately activated carbonyl-group-containing compounds **1a**–**e**, C_1_ synthon **2a**,**b** and hippuric acid (**4**).

**Scheme 2:**
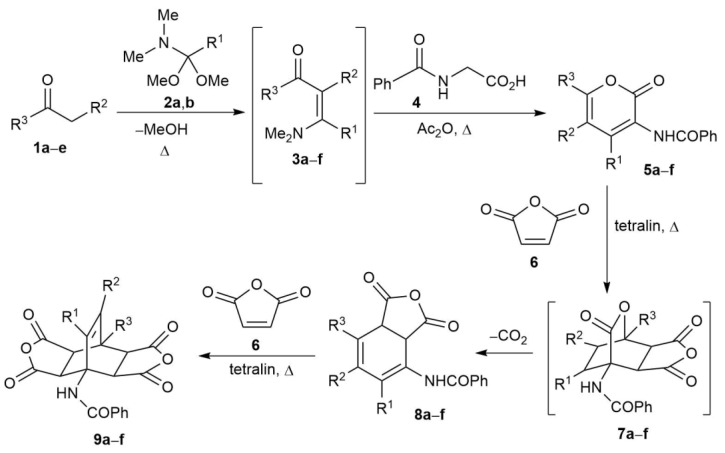
Synthesis of starting bicyclo[2.2.2]octenes **9a**–**f**.

The first step (Scheme 2) [46,47,48,49] of the synthetic route is thus a neat reaction of a suitable carbonyl compound **1a**–**e** possessing an activated α-CH_2_ unit with an appropriate C_1_ synthon (DMFDMA (**2a**, R^1^ = H) in all cases, except for **5b**, where the incorporation of 4-methyl group in the 2*H*-pyran-2-one product requires the use of a different C_1_ synthon, i.e., DMADMA (**2b**, R^1^ = Me)), thus forming *N*,*N*-dimethylaminomethylenes **3a**–**f**. Thereafter, excess of **2** and methanol (formed as the side product) are removed with vacuum distillation and to the viscous remainder, containing intermediates **3a**–**f**, hippuric acid (**4**) and acetanhydride are added. Upon heating, a molecule of water is eliminated from **4**, thus forming its cyclic derivative (2-phenyloxazol-5(4*H*)-one), which condenses with **3a**–**f** to produce an intermediate which is transformed via ring-opening/ring-closing steps to the desired 2*H*-pyran-2-ones **5a**–**f**. Compounds **5** are isolated as solids by vacuum filtration and purified by crystallization.

Next comes the crucial step of building the fused bicyclo[2.2.2]octene skeleton. The approach used was based on our previous experience [50] where two consecutive Diels–Alder cycloadditions of maleic anhydride (or *N*-substituted maleimides) provided the desired skeleton in high yields and with complete stereoselectivity. 2*H*-Pyran-2-ones **5a**–**f** were thus reacted with maleic anhydride (**6**) in refluxing tetralin, yielding bicyclo[2.2.2]octenes **9a**–**f** containing strategically positioned fused succinic anhydride rings. In the first step of this transformation (**5** → **9**), maleic anhydride (**6**), acting as an electron-deficient dienophile, reacts with **5a**–**f** in a normal electron demand Diels–Alder reaction, thus forming carbon-dioxide-bridged bicyclo[2.2.2]octenes **7a**–**f**, which are thermally labile. Under the applied reaction conditions, a spontaneous elimination of carbon dioxide from **7a**–**f** takes place via a retro-hetero-Diels–Alder reaction, thus yielding cyclohexadiene intermediates **8a**–**f**, which react with a second molecule of dienophile **6**, forming the desired bicyclo[2.2.2]octenes **9a**–**f**. Products **9** are isolated by vacuum filtration and purified by crystallization.

**Scheme 3:**
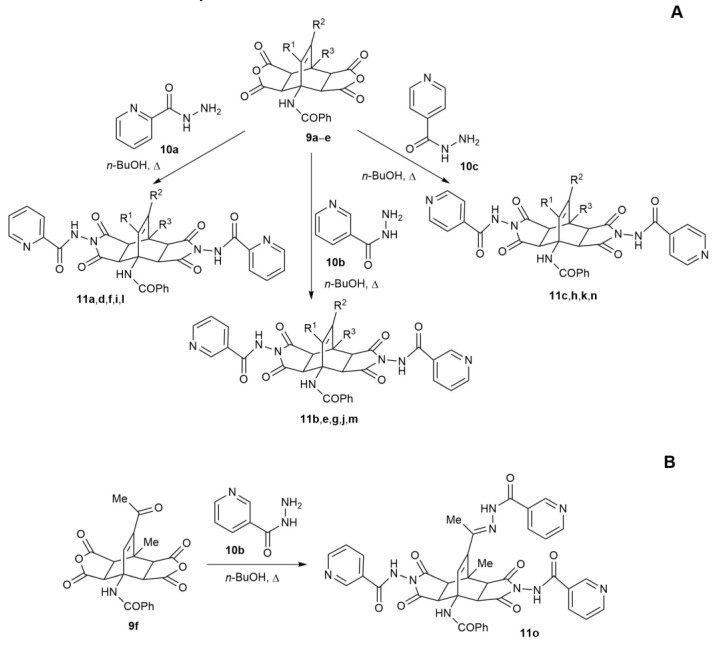
(**A**) Synthesis of the final adducts **11a**–**n**. (**B**) Synthesis of the final adduct **11o**.

The last step (Scheme 3) is composed of a straightforward nucleophilic attack of the terminal amino group of regioisomeric pyridylcarbohydrazides **10a**–**c** on the succinic anhydride moiety of the bicyclo[2.2.2]octenes **9a**–**f**, analogously to our previous investigations [28,51]. This reaction takes place in closed thick-walled glass tubes at 160 °C in *n*-butanol as a suitable solvent, which enables easy isolation of crude products **11a**–**o** upon completion of the reaction. Cooling of the reaction mixtures namely triggers precipitation of ethenopyrrolo[3,4-*f*]isoindoles **11a**–**o** possessing the required bicyclo[2.2.2]octene skeletons, which are collected by vacuum filtration and further purified by crystallization from suitable solvents (methanol for **11a**–**c**,**i**–**k**, ethanol for **11d**–**h**,**l**,**m**, ethyl acetate for **11n**, and mixture of acetone/DMF for **11o**). Because starting compound **9f** contains an additional carbonyl group (i.e., R^2^ = -COMe), this one reacts with the hydrazide **10b** as well, so the corresponding hydrazone **11o** is obtained (Scheme 3B), consistent with reactivity described in the literature [51].

According to TLC and ^1^H NMR analyses of crude reaction mixtures, the conversions from **9** to **11** were quantitative (above 98%) already after 3 h of heating at 160 °C with 5% excess of hydrazides **10**. Only in the case of the synthesis of **11e** did the above-mentioned conditions lead to poor conversion (estimated to be only slightly above 60%). Therefore, the increase in the reaction time to 10.5 h with concomitant increase in the amount of **10b** to 2.0 mmol (per 0.5 mmol **9b**; i.e., 100% excess of **10b**) was shown to be necessary to enable quantitative conversion.

All structures were confirmed on the basis of spectroscopic and analytical data, as well as their comparison with the literature data. Structures of the starting bicyclo[2.2.2]octenes **9a**–**f** were previously confirmed by NMR and single-crystal X-ray diffraction studies as exclusively *exo*,*exo* [50]. However, at least in theory, bicyclo[2.2.2]octenes of the type **9** can be formed as four distinctive stereoisomers: as one of two symmetric diastereoisomers (*exo*,*exo*, *endo*,*endo*), or as a pair of asymmetric enantiomers (*exo*,*endo*, *endo*,*exo*). To distinguish between the symmetric and asymmetric pairs is straightforward, as the existence of a symmetry plane in products **9** is immediately noticeable in NMR spectra. Due to the symmetry in **9**, the signals for pairs of protons (3a-H, 4a-H and 7a-H, 8a-H) are, in ^1^H NMR, observed as two doublets for 2H each (with coupling constants typically around 8.6 Hz), instead of four doublets for 1H each, as would be the case for asymmetric bicyclo[2.2.2]octenes [52]. Symmetry plane in the products of the type **9** can also be inferred from ^13^C NMR, where less signals are observed (in comparison with an asymmetric adduct).

With stereostructures of adducts **9** thus unequivocally established, structures **11a**–**o** can also be confirmed as *exo*,*exo* in all cases: (i) there is no mechanistic precedent that would support the isomerization of bicyclo[2.2.2]octene skeleton under the reaction conditions applied for the transformation of **9** into **11** (the nucleophilic attack of NH_2_ group of pyridylcarbohydrazides **10** is taking place only on the anhydride moieties of **9**; thus, all carbons constituting bicyclo[2.2.2]octene skeleton (including 3a-, 4a-, 7a-, and 8a-C) maintain their sp^3^ hybridization throughout the reaction course and no breaking of any bonds on these carbons occurs); (ii) preservation of stereostructures in analogous derivatizations of succinanhydride-fused bicyclo[2.2.2]octenes with primary amines, hydrazine, and its derivatives was already demonstrated [28,51,53]; (iii) NMR data obtained are in agreement with symmetric *exo*,*exo* structures. Namely, for all products **11a**–**o** in ^1^H NMR, two crucial doublets integrated for 2H are observed inside a narrow range of chemical shifts: δ 3.42–4.15 ppm and δ 4.56–4.75 ppm, the former signal for 7a-H and 8a-H and the latter for 3a-H and 4a-H. The range of chemical shifts for the latter set is narrower (only 0.19 ppm), whereas, for the former, it is broader (0.73 ppm). This can be explained by the fact that the environment between 3a-H and 4a-H is less variable (i.e., in all cases, benzoylamino group is bound to 4-C), whereas, between 7a-H and 8a-H, various substituents (R^3^ = Me, Ph, 2-thienly, 2-furyl) are bound to 8-C. Coupling constants for these doublets are in the range of 7.2–8.6 Hz (mean value 8.3 Hz), being consistent with the literature values [28,52] for bicyclo[2.2.2]octene derivatives fused with six-, seven-, or eight-membered carbocycles, where coupling constants for symmetric (*exo*,*exo*) structures were in the range of 8.0–8.6 Hz, while, for asymmetric structures (*endo*,*exo* and *exo*,*endo* racemic mixture), the pair of protons *anti* to the double bond displayed coupling constants between 7.4 and 8.0 Hz and the other pair of protons (*syn* to the double bond) had much larger coupling constants (9.9–10.4 Hz). Comparing the experimental ^1^H NMR data for products **11** and those available in the literature for **9** and related systems [50,52], it is easy to see that the stereostructure of **11** is the same as in **9**, i.e., *exo*,*exo* products are obtained. Retention of stereostructure is obvious also from ^13^C NMR spectra showing that all the structures **9** and **11** are of the same type, i.e., symmetric (as they display a decreased number of signals in comparison with an asymmetric adduct). However, it is worth noting that, in NMR spectra recorded at 29 °C, some signals appear broadened (or doubled) due to the restricted conformation freedom of **11**; as observed previously [28], these signals sharpen (or coalesce) when spectra are recorded at elevated temperature. Additionally, there are no literature data on *endo*,*endo* structures obtained in such Diels–Alder cycloadditions.

### 2.3. Inhibition of SARS-CoV-2 3CL^pro^ Main Protease

**Figure 3:**
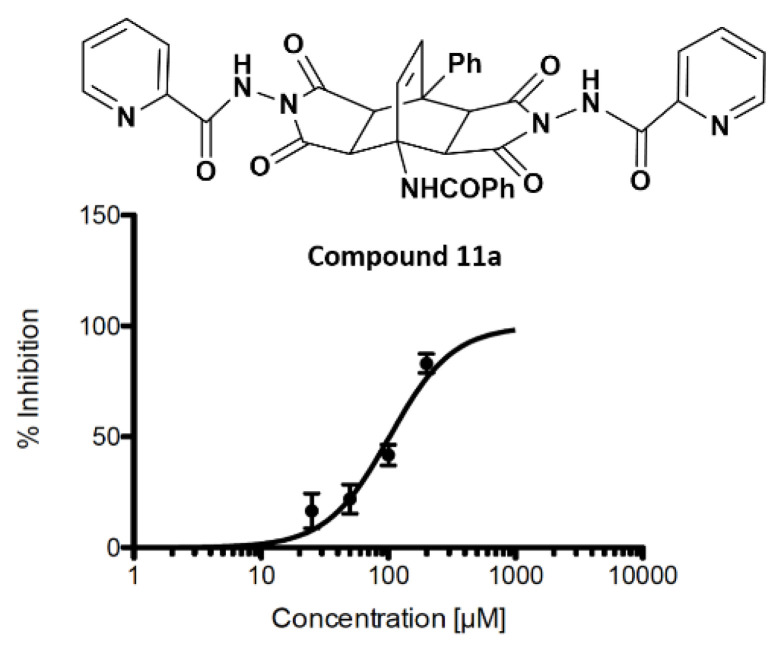
Determination of the IC_50_ value of the SARS-CoV-2 3CL^pro^ inhibition for the most active compound **11a**.

The synthesized fused bicyclo[2.2.2]octenes **11a**–**o** were evaluated for their ability to inhibit SARS-CoV-2 3CL^pro^. To confirm the reproducibility of the measurements, the inhibition experiments were performed several times. The first screening using our fluorescence resonance energy transfer (FRET)-based inhibition assays with isolated SARS-CoV-2 3CL^pro^ was performed at a concentration of 64 μM of each compound. The results indicated that compounds **11a**,**e**,**f**,**i** were candidate hit compounds (Figure S6). Further investigation revealed that some of the compounds had an effect on the measured fluorescence at this concentration. After accounting for this effect, compounds **11a** and **11e** remained as hit candidates (Figures S7 and S8). We subsequently performed assay at compounds’ concentrations of 200, 100, 50, and 25 μM and observed concentration-dependent 3CL^pro^ inhibition. The more active compound **11a** inhibited 3CL^pro^ with an IC_50_ value of 102.2 ± 1.5 μM. The full 3CL^pro^ inhibition was not yet reached at the highest used concentration of the active compound (Figure 3). The results of the inhibition assay for all compounds are shown in Table 2.

Performed 3CL^pro^ inhibition assay confirmed that some representatives of the synthesized fused bicyclo[2.2.2]octenes can noncovalently inhibit the SARS-CoV-2 3CL^pro^ in the micromolar range. Our design strategy has, thus, successfully identified a new rigid scaffold that could be used to develop noncovalent 3CL^pro^ inhibitors. The inhibition activity of compound **11a** is in the comparable micromolar range as determined for nelfinavir (approximately 40 μM) [15]. This result thus constitutes a good point of departure for further optimization endeavors. Although bicyclo[2.2.2]octene skeletons are less frequently encountered among biologically active compounds, there are some previous examples, such as compound mitindomide and its analogues, that display good antitumor activity via inhibition of the human DNA topoisomerase II [54,55,56]. In addition, some of them displayed affinity for the serotonin receptor site, thus acting as potential anxiolytic agents [57]. On the other hand, to the best of our knowledge, there are no literature data on biological activity of 4-aminoethenopyrrolo[3,4-*f*]isoindole derivatives as 3CL^pro^ inhibitors.

### 2.4. Computational Evaluation of Inhibitor Binding to 3CL^pro^

The structure-based molecular design of the fused bicyclo[2.2.2]octene inhibitors was based on the available crystal structure of the SARS-CoV-2 main protease in complex with a covalent peptide-like inhibitor bound to the substrate binding cleft between domains I and II of the 3CL^pro^ enzyme. The binding modes were determined by a standard rigid target/protein docking approach. For a more complex understanding, the flexibility of the protein should be considered when evaluating the proposed binding pose. There are a variety of approaches, such as ensemble docking or the use of molecular simulations [58,59]. With the discovered hit molecule **11a** in hand, dynamic insight into molecular recognition between ligand and protein would also provide valuable information for subsequent compound optimization [60]. Thus, we set up, performed and analyzed an all-atom molecular dynamics (MD) simulation lasting 0.25 µs.

**Figure 4:**
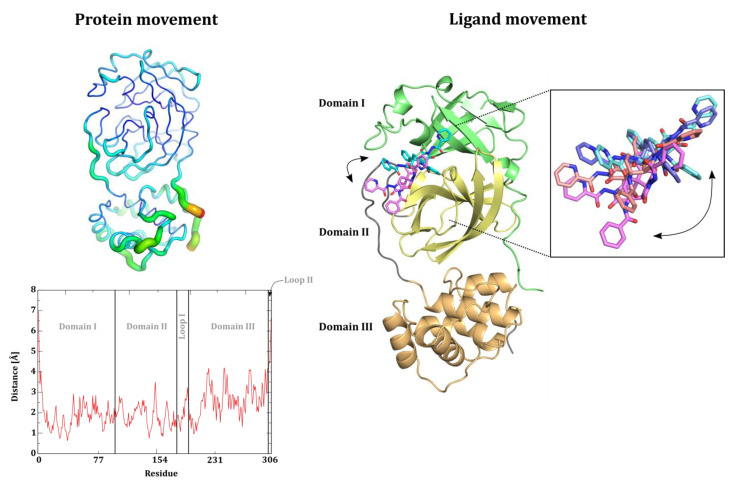
SARS-CoV-2 3CL^pro^ protease (PDB:6LU7) and fused bicyclo[2.2.2]octene **11a** movements observed during the molecular simulation: (right) RMSF heatmap, visualization of the calculated RMSF values, and a corresponding RMSF graph of the protein residues. Both pinpoint the flexibility of the domain III. (left) Detected movement/positions of ligand **11a** in the SARS-CoV-2 3CL^pro^ active site.

We began our analysis of the MD trajectory by calculating the commonly used global geometric parameters. The root-mean-square deviation (RMSD) value of the protein calculated for all Cα atoms (3.48 ± 1.2 Å) shows that the deviation from the initial structure takes place, indicating a general movement of the protein structure (Figure S5). Visualizing the trajectory, we found that the predominant source of structural deviation can be attributed to the movement of the 3CL^pro^ domain III, defined by residues 198 to 303, away from the active site of the protease, which is located between domains I and II. The root-mean-square fluctuations (RMSF) also showed higher values in the domain III region of the protein, while the substrate binding site region showed less fluctuations. Thus, it appears that the flexibility of the 3CL^pro^ domain III does not affect the binding of the ligand (Figure 4, left).

**Figure 5:**
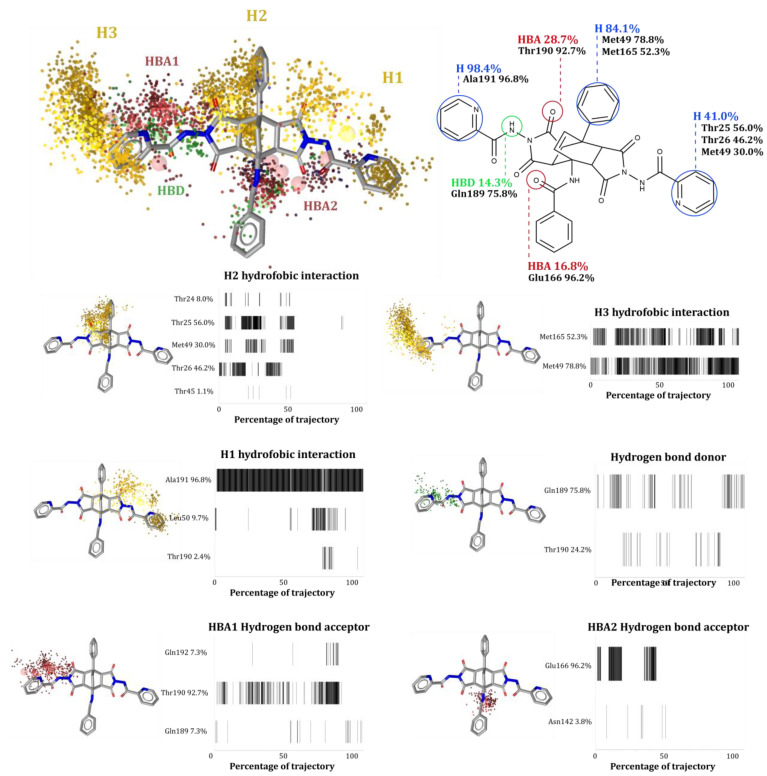
Dynophore model of the bicyclo[2.2.2]octene **11a** in the SARS-CoV-2 3CL^pro^ active site. Top row: (left) 3D dynophore model with point clouds representing the distribution of the obtained superfeatures; (right) detailed 2D schematic depiction of the interaction patterns with amino acid residues and statistical information. Bottom: sequential and statistical schemes of the most important single super-element of the dynophore model. Yellow dots represent hydrophobic element; red ones represent hydrogen bond acceptor features; and green ones hydrogen bond donor features.

RMSD analysis also revealed the flexibility and movement of active ligand **11a** (RMDS = 6.2 ± 2.4 Å, Figure S5). Examination of the MD trajectory revealed two shifts of the compound, the first of which occurs at the 45 ns mark (Figure 5, right). Namely, after the start of the simulation, the ligand was unable to stabilize in the docking orientation between domains I and II, so it realigned its position. Subsequently, the ligand shifted from this position toward the domain II and remained bound to the central region of the domain II after the 120 ns mark (Figure 4, right). The observations derived from the MD simulation reveal that the active site of SARS-CoV-2 3CL^pro^ can accommodate multiple conformations of the compound containing a fused bicyclo[2.2.2]octene.

The observed dynamic behavior in this molecular system prompted us to investigate the ligand interactions with the active site of 3CL^pro^ in more detail. To obtain an overview of the dynamics of the intermolecular interactions, we used a dynamic pharmacophore (dynophore) modeling approach [61]. Here, we can circumvent the limitations of a simple geometric analysis of the intermolecular interactions, such as the measurement of pairwise distances, which often inadequately probe the hydrophobic interactions and H-bonds of the ligand.

The dynophore model obtained for bicyclo[2.2.2]octene **11a** is depicted in Figure 5 and is graphically represented with joint clouds of a particular pharmacophore element (superfeature). The important superfeatures are also shown and analyzed separately. In the dynophore, we can observe the presence of hydrophobic interactions between three of the four H1–H4 moieties (i.e., H1, H2, and H3) of ligand **11a** and hydrophobic residues of the active site of SARS-CoV-2 3CL^pro^, as well as the occurrence of hydrogen bonding between the substrate binding site and the inhibitor. The pharmacophore elements that are part of most superfeatures are quite dispersed, suggesting that the ligand can form productive interactions with the surrounding amino acids despite the observed shift.

Closer examination revealed that the hydrophobic ring H3 is able to form the most focused hydrophobic interaction with Ala191, while the H1 and H2 moieties interact mainly with Met49, albeit less frequently, the latter being a consequence of the positional shift of the compound in the active site of 3CL^pro^ observed during the simulation. In addition, the dynophore isolated three residues possibly involved in hydrogen bonding with the ligand: Glu166, Gln189, and Thr190, which are located around the H3 moiety that forms the most stable hydrogen bonds during the simulation. Interestingly, two residues, Gln189 and Thr190, are located in the loop connecting the 3CL^pro^ domains II and III.

**Figure 6:**
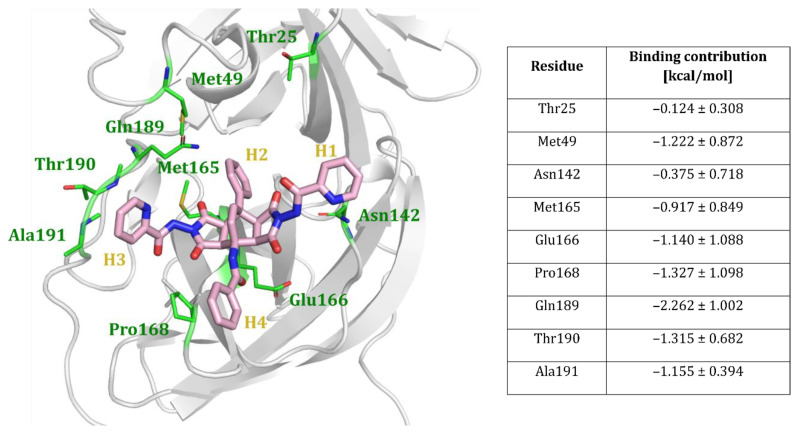
Per-residue energy decomposition of binding free energy calculated by MM/GBSA method. Compound **11a** is shown in an orientation closer to domain II of 3CL^pro^ and depicted in pink, and the residues that contribute the most to its binding are in green.

Next, we extended the geometric analysis of molecular interactions with the assessment of binding thermodynamics by performing the well-established molecular mechanics generalized born surface area (MM/GBSA) endpoint free energy calculations. The estimated binding energy of ligand **11a** was −22.2 ± 6.8 kcal/mol, indicating thermodynamically favorable binding. As part of MM/GBSA, we also performed a per-residue energy decomposition to highlight the residues with the most crucial energetic contributions to the ligand–target interactions (Figure 6).

According to the MM/GBSA per-residue decomposition, the highest energy contribution to binding is associated with the Gln189 residue interaction, followed by contributions from some of the residues already considered important for binding, such as Met49, Thr190, and Ala191. When analyzing the energy contribution of the most important residues, it appears that the protein loop containing amino acid residues 183 to 197 connecting the 3CL^pro^ domains II and III is energetically important for binding.

The energy decomposition results are mostly consistent with those indicated by the dynophore model and both include some residues highlighted by the docking process (i.e., Met49 and Met165); however, some discrepancies remain. The interactions with Thr25 were found to be important in the dynophore, but the energetic analysis assigns little importance to them. This could be related to the stronger interactions that the inhibitor forms with the 3CL^pro^ domain II when it moves away from the initial position. The energetic results also highlighted the importance of Pro168 in binding. Upon visualization of the trajectory, we discovered that, for a significant part of the trajectory, this residue can form stacking-like hydrophobic interaction with the H4 portion of compound **11a**. It is possible that this interaction was not revealed in the dynophore analysis due to this geometry. Both docking and molecular dynamics simulations indicated the importance of the interaction with Gln189. This residue, together with some other residues located on the loop 183–197 connecting the domains II and III, apparently play a crucial role for productive ligand binding to the active site of 3CL^pro^. Molecular simulations highlighted the complexity of molecular recognition and the need for experimental evaluation through structural studies that can provide definitive insights.

## 3. Materials and Methods

### 3.1. Synthetic Procedures and Compounds Characterization Data

Melting points were determined using an automatic OptiMelt MPA100 (Stanford Research System) instrument and are uncorrected. NMR spectra were recorded with a Bruker Avance DPX 300 or Bruker Avance III 500 spectrometer at 29 °C using TMS as the internal standard, at 300 MHz or 500 MHz for ^1^H NMR, respectively, and 75.5 MHz or 125 MHz for ^13^C NMR, respectively. Chemical shifts are provided as ppm values on δ scale and the coupling constants (*J*) are given in Hertz. ^13^C NMR spectra are referenced against the central line of the solvent signal (DMSO-*d*_6_ at 39.5 ppm). IR spectra of compounds as powders were recorded on a Bruker Alpha Platinum ATR FT-IR spectrophotometer. Mass spectra were recorded using an Agilent 6624 Accurate Mass TOF LC/MS spectrometer via ESI ionization. Elemental analyses were performed on a Perkin–Elmer 2400 Series II CHNS/O analyzer.

The starting bicyclo[2.2.2]octenes **9a**–**f** were prepared from appropriately substituted 2*H*-pyran-2-ones **5a**–**f** and maleic anhydride under reflux in tetralin according to the published procedure [50]. The synthesis of starting 2*H*-pyran-2-ones **5a**–**f** starts from appropriate carbonyl compounds **1a**–**f** containing activated CH_2_ groups (i.e., 4-methoxyphenylacetone, 3,4-dimethoxyphenylacetone, or ethyl acetoacetate), C_1_ synthon **2a**,**b** (DMFDMA or DMADMA), and hippuric acid (**4**) upon heating in acetic anhydride, as described previously [46,47,48,49].

Reagents and solvents were used as received from commercial suppliers with purity of 98% or more. Commercially available thick-walled ACE glass tubes closed by a Teflon screw-plug were used.

**2-Pyridylcarbohydrazide (10a)**

Picolinic acid (12.3 g, 0.100 mol) was dissolved in abs. ethanol (80 mL), conc. H_2_SO_4_ (4.0 mL) was added dropwise, and the resulting mixture was refluxed for 20 h, thereafter cooled to room temperature and poured into cold water (50 mL). Aqueous solution of Na_2_CO_3_ was added (approx. 100 mL, concentration 100 g/L) until a pH value of 8 was reached. Undissolved material was filtered off and discarded, and mother liquor extracted with diethyl ether (3 × 40 mL). Volatile components of the combined organic phases were removed in vacuo, yielding brown viscous liquid (ethyl 2-picolinate, 9.62 g, 64%) that was dissolved in ethanol (30 mL), and hydrazine hydrate (6.0 mL) was added. This mixture was refluxed for 12 h; thereafter, all volatile components were removed in vacuo and cold ethanol (2 mL) added to the resulting red oil. Upon cooling, **10a** precipitated and was isolated by filtration. Yellow crystals: yield 5.21 g (38% over both steps), recrystallization from ethanol/water; mp 103–104 °C (lit: 100–102 °C [62]; IR: ν_max_ 3288, 1516, 972, 749 cm^−1^; ^1^H NMR (300 MHz, CDCl_3_): δ 4.10 (s, 2H, NHN*H_2_*), 7.44 (m, 1H), 7.85 (m, 1H), 8.16 (m, 1H), 8.55 (m, 1H) (Py), 9.02 (s, 1H, N*H*NH_2_).

**General procedure for the synthesis of 10b and 10c.** Appropriate ethyl ester (ethyl nicotinoate for **10b**, 20 mL, 0.15 mol; or ethyl isonicotinoate for **10c**, 22 mL, 0.15 mol) was dissolved in ethanol (100 mL). While stirring, hydrazine hydrate (8.75 mL, 0.18 mol) was added slowly; thereafter, the mixture was refluxed (4 h for **10b**, 3 h for **10c**), cooled (8 °C) overnight, and the precipitated product **10b**,**c** isolated by filtration.

**3-Pyridylcarbohydrazide (10b)**

White crystals: yield 18.2 g (89%), recrystallization from ethanol; mp 162–163 °C (lit: 162 °C [63]); IR: ν_max_ 3004, 1670, 1336, 704 cm^−1^; ^1^H NMR (300 MHz, CDCl_3_): δ 4.14 (s, 2H, NHN*H_2_*), 7.58 (br s, 1H, N*H*NH_2_), 8.10 (m, 1H), 8.76 (m, 1H), 8.70 (m, 1H), 8.96 (m, 1H) (Py).

**4-Pyridylcarbohydrazide (10c)**

White crystals: yield 17.9 g (87%), recrystallization from ethanol; mp 174–176 °C (lit: 171–173 °C [64]); IR: ν_max_ 3105, 1661, 1322, 672 cm^−1^; ^1^H NMR (300 MHz, CDCl_3_): δ 4.17 (s, 2H, NHN*H_2_*), 7.58 (br s, 1H, N*H*NH_2_), 8.32 (m, 2H), 8.82 (m, 2H) (Py).

**General procedure for the synthesis of 11a**–**o.** A mixture of the starting bicyclo[2.2.2]octene derivative **9a**–**f** (0.500 mmol), pyridylcarbohydrazide **10a**–**c** (1.10 mmol or 1.65 mmol for the synthesis of **11o**) and *n*-butanol (6 mL) was heated in a closed thick-walled glass tube (15 mL volume) equipped with a magnetic stirring bar for 3 h at 160 °C. Thereafter, the reaction vessel was cooled to room temperature, and the precipitated product was filtered off and washed with water (3 mL). Crude products **11a**–**o** thus obtained were recrystallized.

***N*-[8-Phenyl-2,3,3a,4a,5,6,7,7a,8,8a-decahydro-1,3,5,7-tetraoxo-2,6-bis(2-pyridylcarboamino)-4,8-ethenobenzo[1,2-*c*:4,5-*c*′]dipyrrol-4(1*H*)-yl]benzamide (11a).** White crystals: yield 236 mg (69%), recrystallization from MeOH, mp 263–265 °C; IR: ν_max_ 1790, 1730, 995, 614 cm^−1^; ^1^H NMR (500 MHz, DMSO-*d*_6_): δ 4.07 (d, *J* = 8.3 Hz) and 4.23 (br s) (2H, 7a-H, 8a-H), 4.70 (br d, *J* = 8.3 Hz, 2H, 3a-H, 4a-H), 6.95 (m, 2H), 7.30 (m, 2H), 7.53 (m, 7H), 7.90 (m, 3H), 8.17 (m, 2H), 8.77 (m, 2H), 8.97 (br s, 2H) (2 × Ph, 2 × Py, 9-H, 10-H), 9.04 (s, 1H, N*H*COPh), 11.13 and 11.41 (2 × s, 2H, 2 × N*H*COPy); ^13^C NMR (125 MHz, DMSO-*d*_6_): δ 42.0, 45.8, 47.2, 58.1, 123.8, 126.6, 126.7, 126.9, 127.5, 127.7, 128.1, 129.8, 131.2, 132.0, 135.4, 137.9, 148.5, 153.2, 163.1, 168.4, 170.8, 171.1 (one aromatic signal is hidden); MS (ESI+, TOF): *m/z* 682 ([M+H]^+^). HRMS Calcd for C_37_H_28_N_7_O_7_ (MH^+^): 682.2045. Found: 682.2056.

***N*-[8-Phenyl-2,3,3a,4a,5,6,7,7a,8,8a-decahydro-1,3,5,7-tetraoxo-2,6-bis(3-pyridylcarboamino)-4,8-ethenobenzo[1,2-*c*:4,5-*c*′]dipyrrol-4(1*H*)-yl]benzamide (11b).** White crystals: yield 238 mg (70%), recrystallization from MeOH, mp 278–282 °C; IR: ν_max_ 1792, 1725, 1198, 701 cm^−1^; ^1^H NMR (300 MHz, DMSO-*d*_6_): δ 4.15 (br d, 2H, 7a-H, 8a-H), 4.70 (br d, 2H, 3a-H, 4a-H), 6.84 (d, *J* = 8.9 Hz, 1H) and 7.03 (d, *J* = 8.9 Hz, 1H) (AB, 9-H, 10-H), 7.31–7.92 (m, 12H), 8.17 (br d, 2H), 8.77 (m, 2H), 8.98 (br d, 2H) (2 × Ph, 2 × Py), 9.04 (s, 1H, N*H*COPh), 11.20 (br s, 2H, 2 × N*H*COPy); ^13^C NMR (125 MHz, DMSO-*d*_6_): δ 42.5, 46.2, 47.6, 58.6, 124.3, 127.0, 127.4, 127.9, 128.2, 128.6, 130.3, 131.7, 132.5, 135.9, 138.4, 148.9, 153.7, 163.5, 168.9, 171.3, 171.6 (two aromatic signals are hidden); MS (ESI+, TOF): *m/z* 682 ([M+H]^+^). Anal. Calcd for C_37_H_27_N_7_O_7_ · 3 H_2_O: C, 60.40; H, 4.52; N, 13.33. Found: C, 61.16; H, 4.47; N, 13.47.

***N*-[8-Phenyl-2,3,3a,4a,5,6,7,7a,8,8a-decahydro-1,3,5,7-tetraoxo-2,6-bis(4-pyridylcarboamino)-4,8-ethenobenzo[1,2-*c*:4,5-*c*′]dipyrrol-4(1*H*)-yl]benzamide (11c).** White crystals: yield 245 mg (72%), recrystallization from MeOH, mp 289–293 °C; IR: ν_max_ 1795, 1733, 1286, 699 cm^−1^; ^1^H NMR (300 MHz, DMSO-*d*_6_): δ 4.14 (br d, 2H, 7a-H, 8a-H), 4.70 (br d, 2H, 3a-H, 4a-H), 6.83 (d, *J* = 8.9 Hz, 1H) and 7.02 (d, *J* = 8.9 Hz, 1H) (AB, 9-H, 10-H), 7.30–7.90 (m, 14H), 8.77 (m, 4H) (2 × Ph, 2 × Py), 9.03 (s, 1H, N*H*COPh), 11.24–11.50 (br s, 2H, 2 × N*H*COPy); ^13^C NMR (125 MHz, DMSO-*d*_6_): δ 42.8, 46.2, 47.9, 58.6, 121.7, 127.2, 128.0, 128.2, 128.6, 130.3, 131.7, 132.5, 135.9, 138.2, 138.6, 151.1, 163.5, 168.9, 171.2, 171.5 (one aromatic signal is hidden); MS (ESI+, TOF): *m/z* 682 ([M+H]^+^). Anal. Calcd for C_37_H_27_N_7_O_7_ · 1.5 H_2_O: C, 62.71; H, 4.27; N, 13.84. Found: C, 62.62; H, 3.93; N, 13.74.

***N*-[8-Phenyl-2,3,3a,4a,5,6,7,7a,8,8a-decahydro-10-methyl-1,3,5,7-tetraoxo-2,6-bis(2-pyridylcarboamino)-4,8-ethenobenzo[1,2-*c*:4,5-*c*′]dipyrrol-4(1*H*)-yl]benzamide (11d).** Off-white crystals: yield 215 mg (62%), recrystallization from EtOH, mp 271–274 °C; IR: ν_max_ 1789, 1727, 1488, 780 cm^−1^; ^1^H NMR (300 MHz, DMSO-*d*_6_): δ 2.09 (s, 3H, Me), 4.06 (d, *J* = 8.3 Hz, 2H, 7a-H, 8a-H), 4.71 (d, *J* = 8.3 Hz, 2H, 3a-H, 4a-H), 6.59 (s, 1H, 9-H), 7.28 (m, 2H), 7.42 (m, 2H), 7.51 (m, 2H), 7.64 (m, 4H), 7.85 (m, 3H), 8.02 (m, 4H), 8.68 (m, 2H) (N*H*COPh, 2 × Ph, 2 × Py), 11.09 (br s, 2H, 2 × N*H*COPy); ^13^C NMR (125 MHz, DMSO-*d*_6_): δ 19.5, 42.5, 46.1, 47.7, 60.2, 123.2, 124.3, 127.1, 127.8, 128.2, 128.5, 128.8, 131.9, 135.8, 138.5, 138.8, 148.3, 149.3, 162.6, 168.9, 171.4, 171.6 (two aromatic signals are hidden); MS (ESI+, TOF): *m/z* 696 ([M+H]^+^).

***N*-[8-Phenyl-2,3,3a,4a,5,6,7,7a,8,8a-decahydro-10-methyl-1,3,5,7-tetraoxo-2,6-bis(3-pyridylcarboamino)-4,8-ethenobenzo[1,2-*c*:4,5-*c*′]dipyrrol-4(1*H*)-yl]benzamide (11e).** Off-white crystals: yield 292 mg (84%), recrystallization from EtOH, mp 345–347 °C (with decomposition); IR: ν_max_ 1732, 1670, 1016, 686 cm^−1^; ^1^H NMR (500 MHz, DMSO-*d*_6_): δ 2.11 (s, 3H, Me), 4.06 (br s) and 4.17 (d, *J* = 7.5 Hz) (2H, 7a-H, 8a-H), 4.75 and 4.80 (2×d, *J* = 7.5 Hz, 2H, 3a-H, 4a-H), 6.65 (m, 1H), 7.30 (m, 2H), 7.45 (br s, 2H), 7.54 (m, 6H), 7.86 (m, 3H), 8.17 (m, 2H), 8.77 (m, 2H), 8.97 (br s, 2H) (N*H*COPh, 2 × Ph, 2 × Py, 9-H), 11.13 and 11.40 (2×s, 2H, 2 × N*H*COPy); ^13^C NMR (125 MHz, DMSO-*d*_6_): δ 19.0, 42.3, 45.7, 47.5, 59.8, 123.8, 126.6, 126.7, 126.9, 127.4, 127.8, 128.3, 131.5, 135.5, 138.1, 138.4, 138.8, 148.6, 153.1, 153.2, 163.1, 168.6, 171.0, 171.4; MS (ESI+, TOF): *m/z* 696 ([M+H]^+^). HRMS Calcd for C_38_H_30_N_7_O_7_ (MH^+^): 696.2201. Found: 696.2206.

***N*-[2,3,3a,4a,5,6,7,7a,8,8a-Decahydro-1,3,5,7-tetraoxo-2,6-bis(2-pyridylcarboamino)-8-(2-thienyl)-4,8-ethenobenzo[1,2-*c*:4,5-*c*′]dipyrrol-4(1*H*)-yl]benzamide (11f).** Off-white crystals: yield 248 mg (72%), recrystallization from EtOH, mp 288–291 °C (with decomposition); IR: ν_max_ 1789, 1725, 1213, 611 cm^−1^; ^1^H NMR (300 MHz, DMSO-*d*_6_): δ 3.95 (br s, 2H, 7a-H, 8a-H), 4.66 (d, *J* = 8.5 Hz, 2H, 3a-H, 4a-H), 6.64 (br s, 1H) and 6.79 (d, *J* = 8.8 Hz, 1H) (AB, 9-H, 10-H), 6.95–8.06 (m, 14H), 8.68 (m, 2H) (2-thienyl, Ph, 2 × Py), 9.03 (s, 1H, N*H*COPh), 11.08 (br s, 2H, 2 × N*H*COPy); ^13^C NMR (125 MHz, DMSO-*d*_6_): δ 42.7, 44.6, 49.5, 58.4, 123.2, 126.1, 128.2, 128.5, 130.8, 131.6, 132.4, 135.9, 138.5, 143.5, 148.4, 149.3, 162.7, 168.9, 171.0, 171.2 (three aromatic signals are hidden); MS (ESI+, TOF): *m/z* 688 ([M+H]^+^).

***N*-[2,3,3a,4a,5,6,7,7a,8,8a-Decahydro-1,3,5,7-tetraoxo-2,6-bis(3-pyridylcarboamino)-8-(2-thienyl)-4,8-ethenobenzo[1,2-*c*:4,5-*c*′]dipyrrol-4(1*H*)-yl]benzamide (11g).** Off-white crystals: yield 270 mg (79%), recrystallization from EtOH, mp 259–262 °C (with decomposition); IR: ν_max_ 1792, 1724, 1203, 702 cm^−1^; ^1^H NMR (300 MHz, DMSO-*d*_6_): δ 3.94 (br s, 2H, 7a-H, 8a-H), 4.71 (br s, 2H, 3a-H, 4a-H), 6.82 (d, *J* = 8.8 Hz, 2H, AB, 9-H, 10-H), 7.04 (s, 1H, 2-thienyl), 7.25 (br s, 1H), 7.54 (m, 6H), 7.91 (m, 2H), 8.19 (m, 2H), 8.77 (m, 2H), 8.99 (m, 2H)(2-thienyl, Ph, 2 × Py), 9.02 (s, 1H, N*H*COPh), 11.16 (br s, 1H), 11.43 (br s, 1H) (2 × N*H*COPy); ^13^C NMR (125 MHz, DMSO-*d*_6_): δ 42.8, 44.6, 49.4, 58.4, 124.3, 126.5, 127.0, 127.4, 128.1, 128.6, 131.7, 132.6, 135.9, 143.5, 149.0, 153.7, 163.6, 168.8, 171.1, 171.3 (three aromatic signals are hidden); MS (ESI+, TOF): *m/z* 688 ([M+H]^+^). Anal. Calcd for C_35_H_25_N_7_O_7_S· 2.5 H_2_O: C, 57.37; H, 4.13; N, 13.38. Found: C, 57.56; H, 3.70; N, 13.19.

***N*-[2,3,3a,4a,5,6,7,7a,8,8a-Decahydro-1,3,5,7-tetraoxo-2,6-bis(4-pyridylcarboamino)-8-(2-thienyl)-4,8-ethenobenzo[1,2-*c*:4,5-*c*′]dipyrrol-4(1*H*)-yl]benzamide (11h).** Off-white crystals: yield 265 mg (77%), recrystallization from EtOH, mp 310–312 °C; IR: ν_max_ 1794, 1736, 1209, 782 cm^−1^; ^1^H NMR (300 MHz, DMSO-*d*_6_): δ 4.01 (br s, 2H, 7a-H, 8a-H), 4.72 (br s, 2H, 3a-H, 4a-H), 6.82 (m, 2H, 9-H, 10-H), 6.95–7.95 (m, 12H), 8.78 (m, 2H) (2-thienyl, Ph, 2 × Py), 9.03 (s, 1H, N*H*COPh), 11.29 (br s, 1H), 11.55 (br s, 1H) (2 × N*H*COPy); ^13^C NMR (125 MHz, DMSO-*d*_6_): δ 43.0, 44.7, 49.8, 58.4, 121.7, 126.6, 128.1, 128.6, 131.7, 132.7, 135.9, 138.2, 143.5, 151.1, 163.5, 168.8, 171.0, 171.1 (three aromatic signals are hidden); MS (ESI+, TOF): *m/z* 688 ([M+H]^+^).

***N*-[8-(2-Furyl)-2,3,3a,4a,5,6,7,7a,8,8a-decahydro-1,3,5,7-tetraoxo-2,6-bis(2-pyridylcarboamino)-4,8-ethenobenzo[1,2-*c*:4,5-*c*′]dipyrrol-4(1*H*)-yl]benzamide (11i).** Off-white crystals: yield 228 mg (68%), recrystallization from MeOH, mp 332–334 °C; IR: ν_max_ 1797, 1738, 1211, 785 cm^−1^; ^1^H NMR (300 MHz, DMSO-*d*_6_): δ 3.94 (d, *J* = 8.6 Hz, 2H, 7a-H, 8a-H), 4.66 (d, *J* = 8.6 Hz, 2H, 3a-H, 4a-H), 6.43 (m, 1H), 6.51 (br s, 1H) (2-furyl), 6.60 (d, *J* = 8.8 Hz, 1H) and 6.78 (d, *J* = 8.8 Hz, 1H) (AB, 9-H, 10-H), 7.49–8.02 (m, 12H), 8.69 (m, 2H) (2-furyl, Ph, 2 × Py), 9.05 (s, 1H, N*H*COPh), 11.14 (br s, 2H, 2 × N*H*COPy); ^13^C NMR (125 MHz, DMSO-*d*_6_): δ 42.3, 43.0, 46.5, 58.4, 108.7, 110.9, 123.2, 128.2, 128.5, 129.1, 129.5, 131.6, 132.6, 136.0, 138.5, 142.9, 148.4, 149.3, 151.6, 162.7, 168.9, 171.1, 171.4; MS (ESI+, TOF): *m/z* 672 ([M+H]^+^).

***N*-[8-(2-Furyl)-2,3,3a,4a,5,6,7,7a,8,8a-decahydro-1,3,5,7-tetraoxo-2,6-bis(3-pyridylcarboamino)-4,8-ethenobenzo[1,2-*c*:4,5-*c*′]dipyrrol-4(1*H*)-yl]benzamide (11j).** Off-white crystals: yield 235 mg (70%), recrystallization from MeOH, mp 272–276 °C; IR: ν_max_ 1793, 1731, 1191, 701 cm^−1^; ^1^H NMR (300 MHz, DMSO-*d*_6_): δ 4.04 (d, *J* = 7.2 Hz, 2H, 7a-H, 8a-H), 4.72 (d, *J* = 7.2 Hz, 2H, 3a-H, 4a-H), 6.50 (m, 2H, 2-furyl), 6.71 (d, *J* = 8.8 Hz, 1H) and 6.82 (d, *J* = 8.8 Hz, 1H) (AB, 9-H, 10-H), 7.54 (m, 5H, Ph, 2 × Py), 7.72 (s, 1H, 2-furyl), 7.91 (br d, 2H), 8.19 (br d, 2H), 8.79 (m, 2H), 9.01 (m, 3H) (Ph, 2 × Py, N*H*COPh), 11.18 (br s, 1H), 11.44 (br s, 1H) (2 × N*H*COPy); ^13^C NMR (125 MHz, DMSO-*d*_6_): δ 42.4, 43.1, 46.6, 58.4, 108.9, 111.0, 124.3, 127.0, 127.4, 128.1, 128.6, 129.9, 131.7, 132.9, 135.1, 143.0, 149.0, 151.5, 153.6, 163.6, 168.8, 171.1, 171.5; MS (ESI+, TOF): *m/z* 672 ([M+H]^+^). Anal. Calcd for C_35_H_25_N_7_O_8_ · 1.5 H_2_O: C, 60.17; H, 4.04; N, 14.03. Found: C, 60.33; H, 4.32; N, 14.09.

**N-[8-(2-Furyl)-2,3,3a,4a,5,6,7,7a,8,8a-decahydro-1,3,5,7-tetraoxo-2,6-bis(4-pyridylcarboamino)-4,8-ethenobenzo[1,2-c:4,5-c′]dipyrrol-4(1H)-yl]benzamide (11k).** Off-white crystals: yield 245 mg (73%), recrystallization from MeOH, mp 309–311 °C; IR: νmax 1794, 1740, 1210, 784 cm^−1^; ^1^H NMR (300 MHz, DMSO-d6): δ 4.00 (br d, 2H, 7a-H, 8a-H), 4.72 (br s, 2H, 3a-H, 4a-H), 6.43–6.84 (m, 5H), 7.46–7.94 (m, 10H), 8.79 (m, 4H) (2-furyl, Ph, 2 × Py), 9.02 (s, 1H, NHCOPh), 11.31 (br s, 1H), 11.56 (br s, 1H) (2 × NHCOPy); 13C NMR (125 MHz, DMSO-d6): δ 42.4, 43.1, 46.6, 58.4, 108.9, 111.0, 121.8, 128.1, 128.6, 129.8, 131.7, 132.9, 135.9, 138.2, 138.6, 143.0, 151.1, 163.5, 168.8, 171.0, 171.4; MS (ESI+, TOF): *m*/*z* 672 ([M+H]+).

***N*-[2,3,3a,4a,5,6,7,7a,8,8a-Decahydro-8-methyl-9-(4-methoxyphenyl)-1,3,5,7-tetraoxo-2,6-bis(2-pyridylcarboamino)-4,8-ethenobenzo[1,2-*c*:4,5-*c*′]dipyrrol-4(1*H*)-yl]benzamide (11l).** Off-white crystals: yield 231 mg (64%), recrystallization from EtOH, mp 298–303 °C (with decomposition); IR: ν_max_ 1786, 1734, 1212, 793 cm^−1^; ^1^H NMR (300 MHz, DMSO-*d*_6_): δ 1.76 (s, 3H, Me), 3.62 (d, *J* = 8.6 Hz, 2H, 7a-H, 8a-H), 3.72 (s, 3H, OMe), 4.60 (d, *J* = 8.6 Hz, 2H, 3a-H, 4a-H), 6.50 (s, 1H, 10-H), 7.01 (m, 4H, 4-MeO-C_6_*H_4_*-), 7.55 (m, 5H), 7.89 (m, 2H), 8.24 (m, 2H), 8.81 (m, 2H) (Ph, 2 × Py), 8.94 (s, 1H, N*H*COPh), 9.04 (m, 2H, 2 × Py), 11.23 (br s, 1H), 11.49 (br s, 1H) (2 × N*H*COPy); ^13^C NMR (125 MHz, DMSO-*d*_6_): δ 30.4, 42.0, 42.6, 47.9, 55.5, 58.4, 113.4, 123.4, 127.4, 128.1, 128.5, 130.2, 130.6, 131.5, 136.1, 138.6, 145.8, 148.5, 149.4, 159.1, 162.8, 171.5, 173.3 (2 aromatic signals are hidden); MS (ESI+, TOF): *m/z* 726 ([M+H]^+^).

***N*-[2,3,3a,4a,5,6,7,7a,8,8a-Decahydro-8-methyl-9-(4-methoxyphenyl)-1,3,5,7-tetraoxo-2,6-bis(3-pyridylcarboamino)-4,8-ethenobenzo[1,2-*c*:4,5-*c*′]dipyrrol-4(1*H*)-yl]benzamide (11m).** Off-white crystals: yield 244 mg (67%), recrystallization from EtOH, mp 365–369 °C (with decomposition); IR: ν_max_ 1788, 1726, 1289, 701 cm^−1^; ^1^H NMR (300 MHz, DMSO-*d*_6_): δ 1.76 (s, 3H, Me), 3.58 (d, *J* = 8.6 Hz, 2H, 7a-H, 8a-H), 3.75 (s, 3H, OMe), 4.62 (d, *J* = 8.6 Hz, 2H, 3a-H, 4a-H), 6.49 (s, 1H, 10-H), 7.01 (m, 4H, 4-MeO-C_6_*H_4_*-), 7.55 (m, 5H), 7.89 (br d, 2H), 8.24 (br d, 2H), 8.81 (m, 2H) (Ph, 2 × Py), 8.94 (s, 1H, N*H*COPh), 9.04 (m, 2H, 2 × Py), 11.23 (br s, 1H), 11.49 (br s, 1H) (2 × N*H*COPy); ^13^C NMR (125 MHz, DMSO-*d*_6_): δ 40.9, 42.1, 42.7, 48.0, 58.3, 121.9, 122.4, 123.1, 128.1, 128.6, 131.7, 135.8, 138.3, 138.7, 142.5, 143.1, 150.0, 150.6, 151.1, 156.6, 163.9, 168.6, 171.2, 172.4 (1 aromatic signal is hidden); MS (ESI+, TOF): *m/z* 726 ([M+H]^+^).

***N*-[2,3,3a,4a,5,6,7,7a,8,8a-Decahydro-8-methyl-9-(4-methoxyphenyl)-1,3,5,7-tetraoxo-2,6-bis(4-pyridylcarboamino)-4,8-ethenobenzo[1,2-*c*:4,5-*c*′]dipyrrol-4(1*H*)-yl]benzamide (11n).** Off-white crystals: yield 233 mg (64%), recrystallization from AcOEt, mp 387–390 °C (with decomposition); IR: ν_max_ 1785, 1728, 1288, 1024, 791 cm^−1^; ^1^H NMR (300 MHz, DMSO-*d*_6_): δ 1.76 (s, 3H, Me), 3.57 (br d, 2H, 7a-H, 8a-H), 3.75 (s, 3H, OMe), 4.62 (br d, 2H, 3a-H, 4a-H), 6.48 (s, 1H, 10-H), 6.75–7.92 (m, 14H), 8.81 (m, 2H) (Ph, 2 × Py), 8.93 (s, 1H, N*H*COPh), 11.34 (br s, 1H), 11.59 (br s, 1H) (2 × N*H*COPy); ^13^C NMR (125 MHz, DMSO-*d*_6_): δ 21.2, 42.8, 48.0, 55.5, 58.5, 60.3, 113.7, 121.9, 127.7, 128.1, 128.5, 130.2, 131.6, 136.0, 138.3, 138.7, 145.7, 151.2, 159.2, 163.8, 168.9, 171.3, 173.2; MS (ESI+, TOF): *m/z* 726 ([M+H]^+^).

***N*-[2,3,3a,4a,5,6,7,7a,8,8a-Decahydro-8-methyl-1,3,5,7-tetraoxo-2,6-bis(3-pyridyl-carboamino)-9-(1-(*N*-[3-pyridylcarbo]hydrazonyl)eth-1-yl)-4,8-ethenobenzo[1,2-*c*:4,5-*c*′]di pyrrol-4(1*H*)-yl]benzamide (11o).** Off-white crystals: yield 186 mg (48%), recrystallization from acetone/DMF, mp 284–286 °C; IR: ν_max_ 1787, 1727, 1278, 698 cm^−1^; ^1^H NMR (300 MHz, DMSO-*d*_6_): δ 2.05 (m, 6H, 2 × Me), 3.42 (br s, 2H, 7a-H, 8a-H), 4.56 (br d, 2H, 3a-H, 4a-H), 6.82 (br s, 1H, 10-H), 7.53 (m, 6H), 7.91 (br d, 2H), 8.22 (m, 3H), 8.80 (m, 3H), 9.02 (m, 4H) (N*H*COPh, Ph, 3 × Py), 10.87 (br s, 1H), 11.17 (m, 2H) (3 × N*H*COPy); ^13^C NMR (125 MHz, DMSO-*d*_6_): δ 16.4, 41.7, 42.7, 47.6, 58.3, 123.9, 124.3, 127.2, 127.4, 128.1, 128.5, 131.7, 135.8, 136.0, 136.4, 149.1, 149.5, 153.7, 163.8, 164.0, 168.6, 171.3, 171.5, 172.6, 173.1 (2 aromatic and 1 aliphatic signals are hidden); MS (ESI+, TOF): *m/z* 781 ([M+H]^+^).

### 3.2. FRET-Based Assay of SARS-CoV-2 3CL^pro^ Main Protease Inhibition Activity

The inhibition assay was performed by a fluorescence resonance energy transfer (FRET) method. A fluorogenic peptide, Dabcyl-KTSAVLQ-SGFRKME-Edans, was utilized as a substrate for the hydrolysis of the SARS-CoV-2 3CL^pro^ protease. The fluorescence intensity was monitored with a fluorescence microplate reader (Spark 10M, TECAN). The chosen wavelengths were 336 nm for excitation and 490 nm for emission, with a bandwidth of 20 nm. The hydrolysis reaction was performed with a buffer consisting of 50 mM Tris-HCl pH 7.3, 1 mM EDTA at 30 °C. The inhibitory activity was first measured in a reaction mixture containing 0.2 nM SARS-CoV-2 3CL^pro^ protease, 4 μM fluorogenic substrate, and compounds **11a**–**o** in concentrations of 64 μM. The fluorescence was monitored every 1 min for a sufficient time period, 60 min. For the most promising compounds **11a** and **11e**, we further performed the above-described assay at compounds´ concentrations of 200, 100, 50, and 25 μM. All experiments were repeated several times.

### 3.3. Molecular Docking Calculations and Binding Site Analysis Using Molecular Probes

Molecular docking was performed using the GOLD software [24] and available SARS-CoV-2 3CL^pro^ main protease X-ray structure (PDB: 6LU7) [8]. The active site was defined as a 6 Å radius around the co-crystallized covalently bound ligand, which was removed from the active site. Hydrogen atoms were added to the protein, and all water molecules were removed. Each molecule was docked into the defined active site by applying the following parameters of the GOLD genetic algorithm (GA): population size = 100, selection pressure = 1.1, no. of operations = 100,000, no. of islands = 5, niche size = 2, migrate = 10, mutate = 95, crossover = 95. GoldScore scoring function was used to assess the favorability of the generated docking solutions. These docking settings were used for the molecular docking calculations of clinically used HIV protease inhibitors (Figure 1), as well as designed bicyclo[2.2.2]octenes **11a**–**n**. Initial 3D conformations of all compounds were generated in ChemBio3D and geometrically optimized by applying the MMFF94 force field. Docking calculations were subsequently visualized and analyzed in LigandScout [22].

LigandScout was also used for the Apo Site Grid analysis [65] using default settings. During this task, various molecular probes (i.e., hydrogen bond acceptor, hydrogen bond donor, positive ionizable, negative ionizable, hydrophobic probes, etc.) scanned SARS-CoV-2 3CL^pro^ active site to generate contours of the corresponding molecular interaction fields (MIFs).

### 3.4. Molecular Dynamics Simulations

For the MD simulation of the SARS-CoV-2 3CL^pro^–ligand **11a** complex, which was obtained with the molecular docking, we first started with the parametrization of the ligand. The partial charges of the ligand were obtained by performing a population analysis according to the Merz–Kollman scheme on the geometry-optimized structure of **11a** at the Hartree–Fock level using the 6–31 G(d) basis set. For the QM optimization, Gaussian 16 was used [66]. RESP charges were generated with the Antechamber module of Amber18 [67]. The remaining ligand’s force field parameters were obtained with Antechamber module, using as input bond lengths and bond angles obtained from the optimized geometries. The General Amber Force Field of second generation (gaff2) was used for the ligand description and the parametrized ligand information is provided in Table S1 [68].

The simulated system was solvated using TIP3P-type water molecules [69] in a cubic box with at least 10 Å from the solute to the edge of the box. Neutral charge of the system was achieved by adding 4 Na^+^ ions. The final system contained approximately 92,400 atoms. Amber14SB force field was used for SARS-CoV-2 3CL^pro^ protein [70] and gaff2 for the ligand [68]. Systems were submitted to an energy minimization of 10,000 steps applying steepest descent, followed by 20,000 steps of conjugate gradient optimization method. This was followed by an NVT equilibration in 4 runs, each 10,000 steps with a time step of 2 fs, with gradually releasing constrain on the protein. Namely, the force constant for the first run was 100 kcal mol^−1^ Å^−2^, second 60 kcal mol^−1^ Å^−2^, third 30 kcal mol^−1^ Å^−2^ and the fourth run was without restraint. This was followed by NPT equilibration in 2 runs: each 100,000 steps, with a time step of 2 fs; in the first run, the SARS-CoV-2 M^pro^ was constrained with the force constant of 20 kcal mol^−1^ Å^−2^ and, in the second run, no constraint was applied. During the NVT equilibration, the systems were gently heated to reach the target temperature of 300 K, controlled by the Langevin thermostat. During the NPT equilibration, the pressure was maintained at 1 bar using the Berendsen barostat. Particle mesh Ewald [71] was applied to treat long-range electrostatics and periodic boundary conditions were applied. SHAKE algorithm [72] was utilized to constrain all bond lengths involving hydrogen atoms to achieve a time step of 2 fs. A total of 0.25 μs of the production MD simulation was then performed using Amber18 cuda program.

#### Analysis of the MD Trajectories

Trajectory obtained during the production stage of the MD simulation was inspected and analyzed using the following tools. Cpptraj module of Ambertools 20 [73] was used to calculate the RMSD and RMSF values. Inhibitor interaction was evaluated energetically by MM/GBSA method [74]. Visualization of the results was conducted using Visual Molecular Dynamics (VMD) [75] and PyMOL [76] softwares.

(a) Cα RMSD and RMSF calculations

Cα RMSD and RMSF analyses were performed on the whole trajectory using Cpptraj module. The RMSD and RMSF values were calculated referring to the initial structure of the protein and ligand, respectively.

(b) MM/GBSA binding free energy calculations

Binding free energy calculations of the protein–ligand complex were performed using the MM/GBSA [76] method included in the AmberTools 20 software suite [75]. Calculations were performed on 1025 snapshots of the MD simulation. We used the generalized Born IGB method 5, and 0.100 M salt concentration. We also performed per-residue decomposition to evaluate the energy contributions of residues to binding.

(c) Dynamic pharmacophore model calculations

Dynophore models were generated using the DynophoreApp v.01 developed in the Molecular Design Lab at Freie Universität (FU), Berlin [77]. A total of 1000 equidistant MD frames of SARS-CoV-2 3CLpro main protease in complex with 11a bound to the substrate-binding cleft located between domains I and II 3CLpro were used for dynophore model generation [78]. These calculations were performed at FU cluster in Berlin and subsequently analyzed and visualized in LigandScout [22].

## 4. Conclusions

The recent emergence of the SARS-CoV-2 virus, responsible for the global COVID-19 pandemic, requires the rapid development of novel antiviral drugs, which, alongside vaccines, are the second cornerstone of effective treatment. Drug repurposing often enables rapid identification of potential drug candidates and selection of molecular scaffolds possessing high functionalization capabilities, thus playing a key role in obtaining optimal preclinical candidates. In our study, we started from the clinically used HIV-1 protease inhibitors, which have been shown to inhibit the replication of SARS-CoV-2 virus and target its major protease 3CL^pro^, one of the most promising antiviral targets of SARS-CoV-2. Using the structure-based design paradigm, we designed and synthesized a series of rigid bicyclo[2.2.2]octenes **11a**–**o** fused to *N*-substituted succinimides to test whether this core scaffold could support the design of noncovalent 3CL^pro^ inhibitors. It was expected that such a rigid scaffold would successfully replace the predominantly flexible scaffold of peptidomimetic HIV-1 protease inhibitors while maintaining the hydrophobic pharmacophore pattern observed for these active molecules. The inhibition assays performed confirmed that some compounds can noncovalently inhibit the SARS-CoV-2 main protease. The 3CL^pro^ inhibitory activity of the most active compound **11a** is in the comparable micromolar range as that determined for nelfinavir, which is a good starting point for further optimization. In this regard, further evaluation of the inhibition mechanism, for example, through kinetic studies, could yield useful information to guide the development of this class of compounds. Molecular simulations of the 3CL^pro^–ligand complex revealed dynamic molecular recognition and flexibility of the ligand, as well as the 3CL^pro^ target itself. The ligand interacted preferentially with active site residues that are part of the protein domain II, as well as with residues of the loop connecting the domains II and III. From an energetic point of view, these appear to play an important role in successful molecular recognition.

Finally, it should be mentioned that covalent inhibitors of the SARS-CoV-2 3CL^pro^ protease, such as the compound PF-07321332, which is under clinical investigation along with ritonavir, have reached the end of clinical trials [79]. There are obvious pharmacological advantages of the covalent inhibitors, such as higher potency and longer duration of action [23]. Nevertheless, the non-covalent inhibitors, with their less pronounced chemical reactivity, will most likely provide another line of defense to combat SARS-CoV-2 infections [80]. Concurrent use with a covalent inhibitor could also be envisaged, as this could delay the development of resistance and increase therapeutic efficacy. We hope that the discovered fused bicyclo[2.2.2]octene scaffold will contribute to the ongoing intensive drug design efforts by providing another starting point for the development of non-covalent SARS-CoV-2 3CL^pro^ protease inhibitors. Moreover, the demonstrated utility of this rigid scaffold could stimulate the development of biologically active molecules containing this skeleton also for other drug targets.

## Figures and Tables

**Table 1:** Reaction conditions and yields for the synthesis of **11a**–**o** **^a^**.

Entry	Starting Bicyclo[2.2.2]octenes 9				Hydrazide 10	*n*(10) in mmol	Product 11	Yield (%) ^b^
	R^1^	R^2^	R^3^					
**1**	H	H	Ph	**9a**	**10a**	2.0	**11a**	69
**2**	H	H	Ph	**9a**	**10b**	2.0	**11b**	70
**3**	H	H	Ph	**9a**	**10c**	2.0	**11c**	72
**4**	Me	H	Ph	**9b**	**10a**	2.0	**11d** ^ c^	62
**5**	Me	H	Ph	**9b**	**10b**	2.0	**11e** ^ c^	84
**6**	H	H	2-thienyl	**9c**	**10a**	1.5	**11f**	72
**7**	H	H	2-thienyl	**9c**	**10b**	1.5	**11g**	79
**8**	H	H	2-thienyl	**9c**	**10c**	1.5	**11h**	77
**9**	H	H	2-furyl	**9d**	**10a**	1.5	**11i**	68
**10**	H	H	2-furyl	**9d**	**10b**	1.5	**11j**	70
**11**	H	H	2-furyl	**9d**	**10c**	1.5	**11k**	73
**12**	H	4-MeO-C_6_H_4_-	Me	**9e**	**10a**	1.1	**11l**	64
**13**	H	4-MeO-C_6_H_4_-	Me	**9e**	**10b**	1.1	**11m**	67
**14**	H	4-MeO-C_6_H_4_-	Me	**9e**	**10c**	1.1	**11n**	64
**15**	H	COMe	Me	**9f**	**10b**	1.65	**11o** ^ d^	48

^a^ Reaction conditions: bicyclo[2.2.2]octenes **9a**–**f** (0.5 mmol), hydrazides **10a**–**c** and *n*-butanol (6 mL) heated for 9 h at 160 °C in a closed vessel. ^b^ Isolated yield. All products were thereafter purified by recrystallization from methanol (**11a**–**c**,**i**–**k**), ethanol (**11d**–**h**,**l**,**m**), ethyl acetate (**11n**), or acetone/DMF (**11o**) to achieve purity above 98% as determined by ^1^H NMR analyses. ^c^ Reaction time 10.5 h. ^d^ **11o**: R^2^ = -C(Me)=NNH-CO-(3-pyridyl).

**Table 2:** Results of the inhibition assay for fused bicyclo[2.2.2]octene **11a**–**o ^a^**.

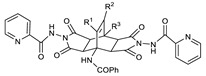				
	**R^1^**	**R^2^**	**R^3^**	**IC_50_ [** **μM]**
**11a**	H	H	Ph	102.2
**11d**	Me	H	Ph	<1000
**11f**	H	H	2-thienyl	<1000
**11i**	H	H	2-furyl	<1000
**11l**	H	4-MeO-C_6_H_4_-	Me	<1000
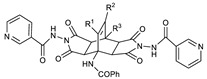				
	**R^1^**	**R^2^**	**R^3^**	
**11b**	H	H	Ph	<1000
**11e**	Me	H	Ph	~200.0
**11g**	H	H	2-thienyl	<1000
**11j**	H	H	2-furyl	<1000
**11m**	H	4-MeO-C_6_H_4_-	Me	<1000
**11o**	H	-C(Me)=NNH-CO-(3-pyridyl)	Me	<1000
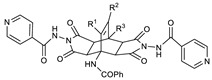				
	**R^1^**	**R^2^**	**R^3^**	
**11c**	H	H	Ph	<1000
**11h**	H	H	2-thienyl	<1000
**11k**	H	H	2-furyl	<1000
**11n**	H	4-MeO-C_6_H_4_-	Me	<1000

^a^ IC50 value > 1000 μM was asigned to all compounds that did not exhibit significant 3CL^pro^ inhibition activity.

## Data Availability

Data is contained within the article and supplementary material.

## Supplementary Materials

The following additional information concerning this manuscript is available online at https://www.mdpi.com/article/10.3390/ph15050539/s1, Figure S1: SARS-CoV-2 3CL^pro^ Main Protease. Calculated molecular interaction fields using hydrophobic ((**A**); yellow), hydrogen bond acceptor ((**B**); red) and hydrogen bond donor ((**C**); green) probes, with the bound ligand N3 shown (PBD: 6LU7); Figure S2. Alignment of docked conformations of HIV-1 protease inhibitors atazanavir (orange), lopinavir (light blue), nelfinavir (green) and ritonavir (violet). The compounds contain four hydrophobic moieties that can fit into four sub-pockets of the 3CL^pro^ active site (PDB: 6LU7); Figure S3: Ligand shift during the molecular dynamics simulation (left) Ligand **11a** in its initial docked position located between domains I and II of 3CL^pro^, (right) Shifted position of **11a** towards the 3CL^pro^ domain II after the 120 ns MD mark. (PDB: 6LU7); Figure S4: Comparison of the experimental conformation of covalent inhibitor bound in the 3CL^pro^ Xray structure and docked position of the ligand **11a** (PDB: 6LU7); Figure S5: (left) Time-dependent RMSD of the 3CL^pro^ protein (Cα atoms) (right), Time-dependent RMSD of the ligand **11a**; Figure S6: Experiments with 3CL^pro^ protease **3CL(+)** fluorescence intensity on *y*-axis is calculated from the value of F(t) – F(0). 64 μM concentration of bicyclo[2.2.2]octenes **11a**–**o** is used and *x*-axis is time in minutes; Figure S7: Experiments without 3CL^pro^ protease **3CL(-)** Background fluorescence intensity (*y*-axis) 64 μM concentration of bicyclo[2.2.2]octenes 11a–o is used and *x*-axis is time in minutes; Figure S8: Data showing the difference between **3CL(+)** and **3CL(-)** 64 μM concentration of bicyclo[2.2.2]octenes 11a–o is used and *x*-axis is time in minutes; Table S1: Atom types and partial atomic charges for compound **11x** from the General Amber Force Field (gaff).
