# Supplementary material for: Design, Synthesis and Evaluation of Fused Bicyclo[2.2.2]octene as a Potential Core Scaffold for the Non-Covalent Inhibitors of SARS-CoV-2 3CLpro Main Protease
Source: Pharmaceuticals (Basel). 2022 Apr 27;15(5):539. doi: 10.3390/ph15050539 (PMC9145702; doi:10.3390/ph15050539)
Supplement: Supplementary file 1 [file pharmaceuticals-15-00539-s001.zip › pharmaceuticals-1693107-supplementary.pdf]

SUPPLEMENTARY INFORMATION:

Design, synthesis and evaluation of fused bicyclo[2.2.2]octene as a potential core scaffold for the non-covalent inhibitors of SARS-COV-2 $3CL^{pro}$ main protease

Barbara Herlah[a,b] Andrej Hoivik[c], Luka Jamšek[c], Katja Valjavec[a], Norio Yamamoto[d], Tyuji Hoshino[e], Krištof Kranjc[c]*, Andrej Perdih[a,b]*

[a]*National Institute of Chemistry, Hajdrihova ulica 19, SI-1000 Ljubljana, Slovenia*

[b]*Faculty of Pharmacy, University of Ljubljana, Aškerčeva 7, SI-1000 Ljubljana, Slovenia*

[c]*Faculty of Chemistry and Chemical Technology, University of Ljubljana, Večna pot 113, SI-1000 Ljubljana, Slovenia*

[d] *Department of Microbiology, Tokai University School of Medicine, 143 Shimokasuya, Isehara, Kanagawa 259-1193, Japan*

[e] *Graduate School of Pharmaceutical Sciences, Chiba University, 1-8-1 Inohana, Chuo-ku, Chiba 260-8675, Japan*

*** Corresponding authors:**

Krištof Kranjc: **kristof.kranjc@fkkt.uni-lj.si**
Andrej Perdih: **andrej.perdih@ki.si**

1. Molecular interaction fields (MIFs) in the SARS-Cov-2 3CL^{pro} main protease active site

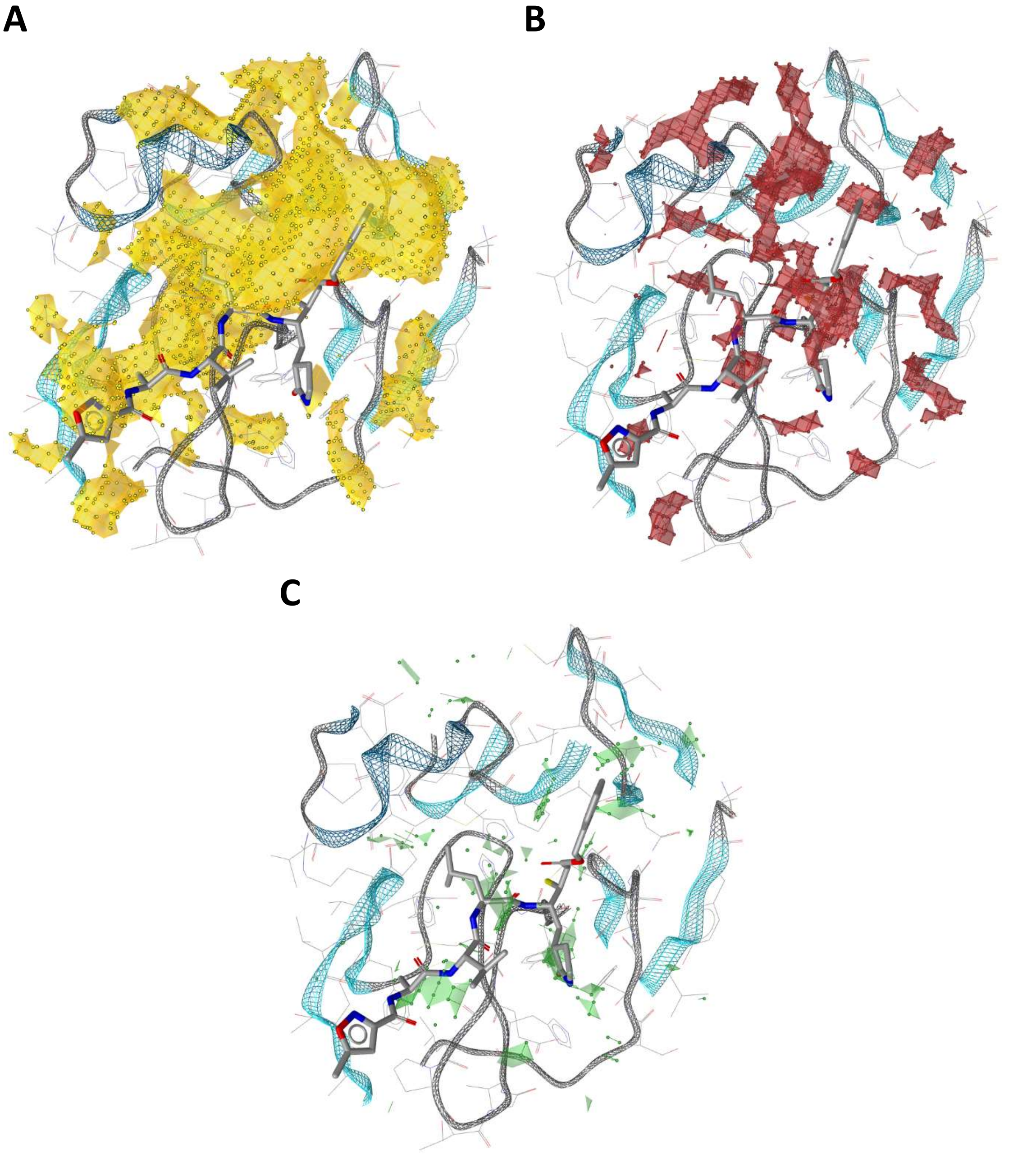

Figure S1. Sars-Cov-2 3CL^{pro} Main Protease. Calculated molecular interaction fields using hydrophobic ((**A**); yellow), hydrogen bond acceptor ((**B**); red) and hydrogen bond donor ((**C**); green) probes, with the bound ligand N3 shown (PBD: 6LU7).

2. Molecular docking calculations of HIV-1 protease inhibitors

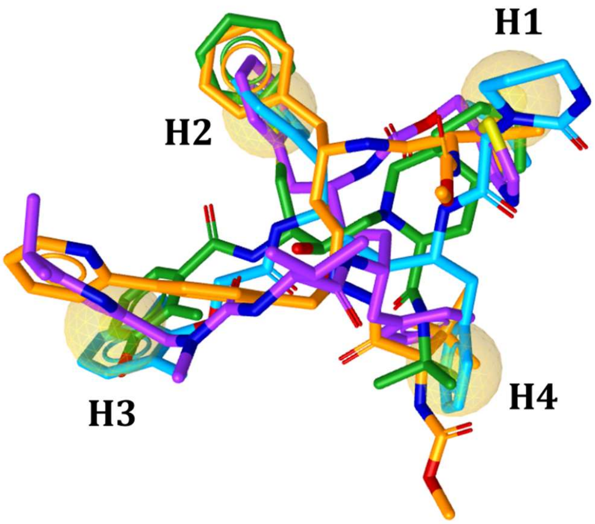

Figure S2. Alignment of docked conformations of HIV-1 protease inhibitors atazanavir (orange), lopinavir (light blue), nelfinavir (green) and ritonavir (violet). The compounds contain four hydrophobic moieties that can fit into four sub-pockets of the 3CL^{pro} active site (PDB: 6LU7).

3. Ligand docking position and ligand movement during molecular simulation

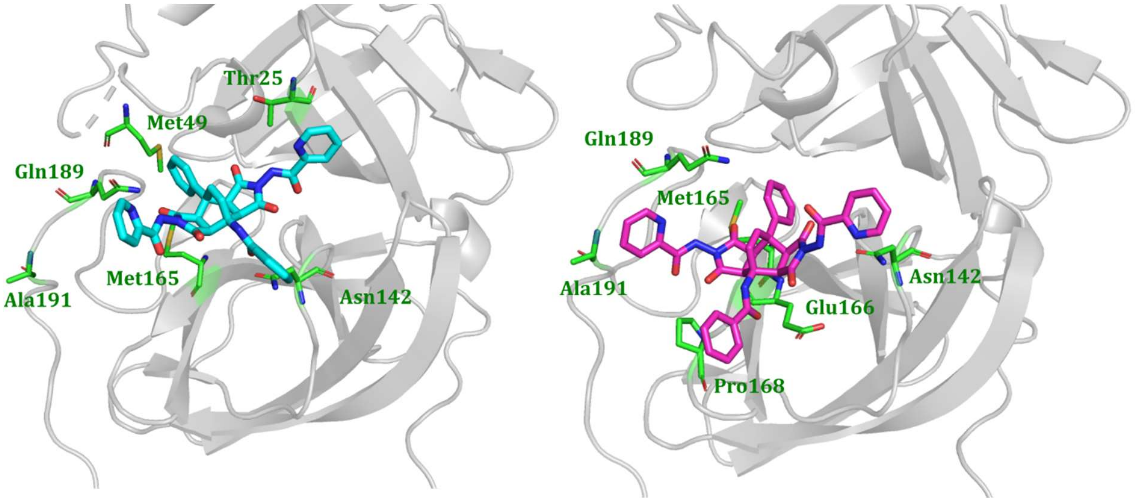

Figure S3. Ligand shift during the molecular dynamics simulation (left) Ligand **11a** in its initial docked position located between domains I and II of 3CL^{pro}, (right) Shifted position of **11a** towards the 3CL^{pro} domain II after the 120 ns MD mark. (PDB: 6LU7) .

4. Comparison with x-ray covalent inhibitor

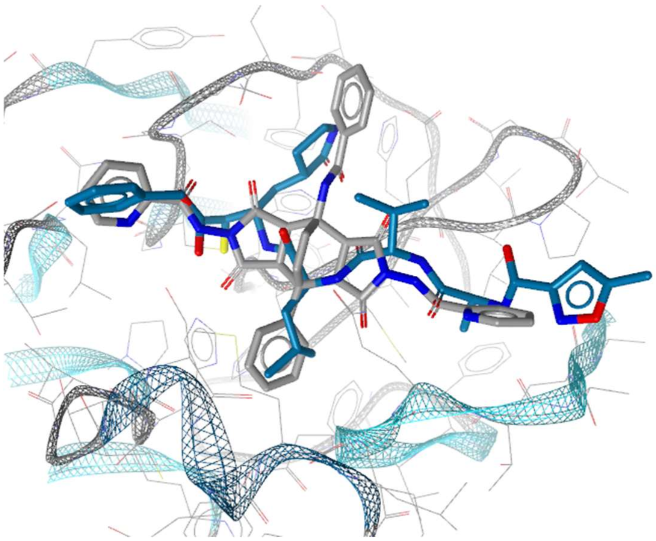

Figure S4. Comparison of the experimental conformation of covalent inhibitor bound in the 3CL^{pro} X-ray structure and docked position of the ligand **11a** (PDB: 6LU7) .

5. Analysis of molecular dynamics simulation

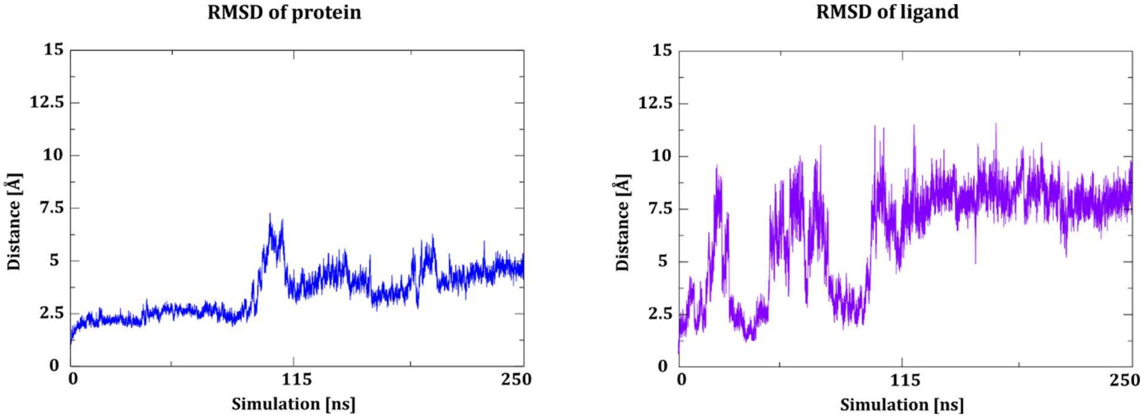

Figure S5. (left) Time-dependent RMSD of the 3CL^{pro} protein (Cα atoms) (right), Time-dependent RMSD of the ligand **11a**.

6. Screening of SARS-CoV-2 3CL^{pro} main protease inhibition

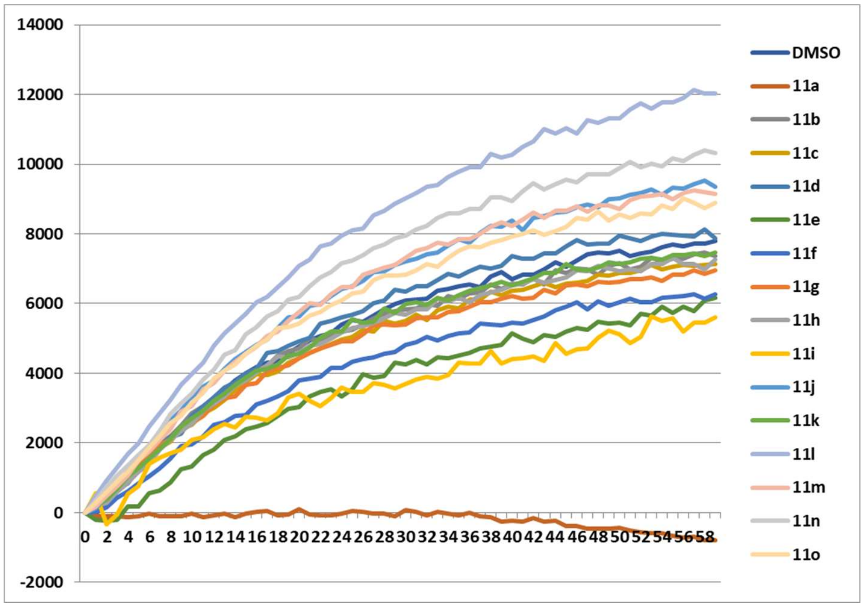

Figure S6. Experiments with 3CL^{pro} protease **3CL(+)** fluorescence intensity on y-axis is calculated from the value of F(t) – F(0). 64 µM concentration of bicyclo[2.2.2]octenes **11a**–**o** is used and x-axis is time in minutes.

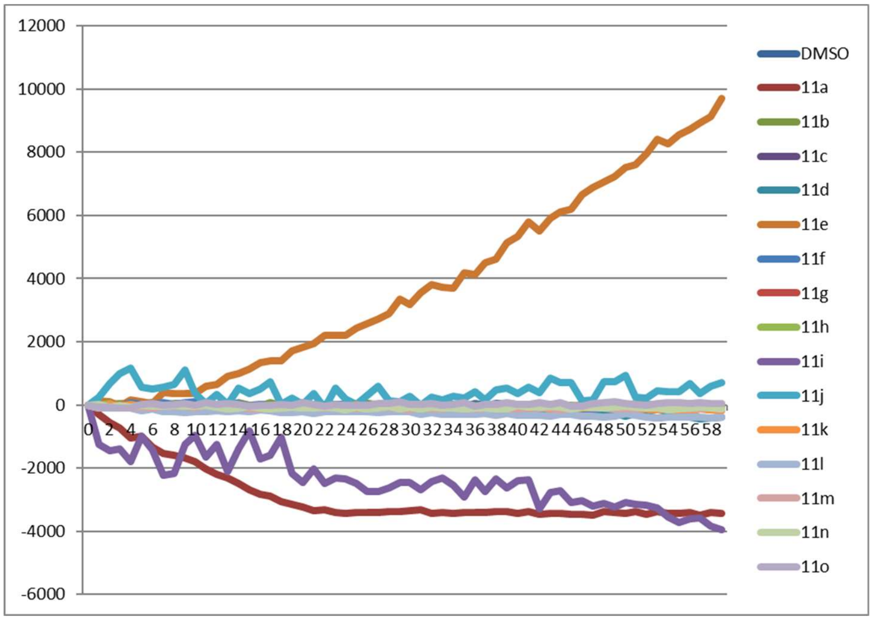

Figure S7. Experiments without 3CL^{pro} protease **3CL(-)** Background fluorescence intensity (y-axis) 64 µM concentration of bicyclo[2.2.2]octenes **11a**–**o** is used and x-axis is time in minutes.

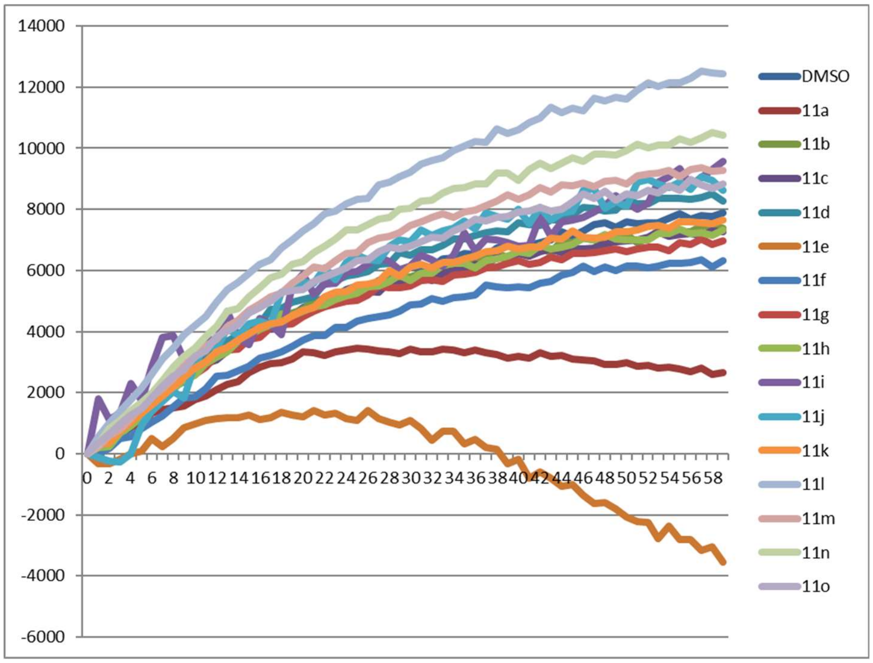

Figure S8. Data showing the difference between **3CL(+)** and **3CL(-)** 64 µM concentration of bicyclo[2.2.2]octenes **11a**–**o** is used and x-axis is time in minutes.

7. Partial charges and atom types of the simulated compound 11a

Table S1: Atom types and partial atomic charges for compound **11x** from the General Amber Force Field (gaff).

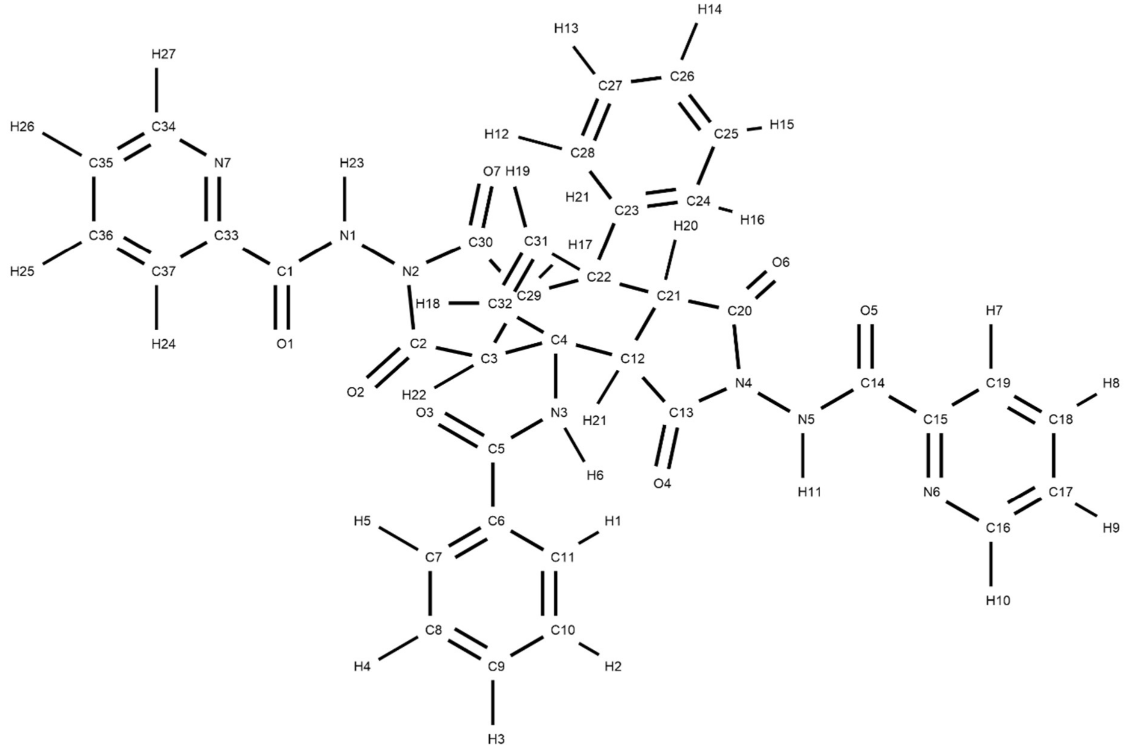

Name	Type	q	Name	Type	q	Name	Type	q
**C10**	ca	-0,15539	**C1**	c	0,650584	**H14**	ha	0,12667
**C11**	ca	-0,09455	**O1**	o	-0,56292	**H15**	ha	0,131839
**H1**	ha	0,141117	**C33**	ca	0,395204	**H16**	ha	0,148091
**H2**	ha	0,136021	**N7**	nb	-0,59114	**C21**	c3	-0,34489
**C9**	ca	-0,08345	**C34**	ca	0,35777	**C20**	c	0,695319
**H3**	ha	0,126235	**C35**	ca	-0,38728	**O6**	o	-0,52909
**C8**	ca	-0,15539	**C36**	ca	0,134943	**H20**	hc	0,077115
**H4**	ha	0,136021	**C37**	ca	-0,36656	**C12**	c3	-0,05793
**C7**	ca	-0,09455	**H24**	ha	0,176752	**H21**	hc	0,122113
**H5**	ha	0,141117	**H25**	ha	0,10953	**C13**	c	0,550358

**C6**	ca	-0,1197	**H26**	ha	0,173901	**O4**	o	-0,50033
**C5**	c	0,82655	**H27**	h4	0,056608	**N4**	n	-0,08768
**O3**	o	-0,60723	**H23**	hn	0,340706	**N5**	n	-0,56424
**N3**	n	-0,8271	**C30**	c	0,695319	**H11**	hn	0,340706
**H6**	hn	0,324279	**O7**	o	-0,52909	**C14**	c	0,650584
**C4**	c3	0,454682	**C29**	c3	-0,34489	**O5**	o	-0,56292
**C32**	c2	-0,28834	**H17**	hc	0,077115	**C15**	ca	0,395204
**C31**	c2	-0,30281	**C22**	c3	0,38365	**N6**	nb	-0,59114
**H19**	ha	0,160165	**C23**	ca	0,013663	**C16**	ca	0,35777
**H18**	ha	0,219125	**C24**	ca	-0,17875	**H10**	h4	0,056608
**C3**	c3	-0,05793	**C25**	ca	-0,1207	**C17**	ca	-0,38728
**H22**	hc	0,122113	**C26**	ca	-0,11945	**H9**	ha	0,173901
**C2**	c	0,550358	**C27**	ca	-0,1207	**C18**	ca	0,134943
**O2**	o	-0,50033	**C28**	ca	-0,17875	**H8**	ha	0,10953
**N2**	n	-0,08768	**H12**	ha	0,148091	**C19**	ca	-0,36656
**N1**	n	-0,56424	**H13**	ha	0,131839	**H7**	ha	0,176752

8. Selected examples of 1H and 13C NMR spectra: compounds 11a and 11e

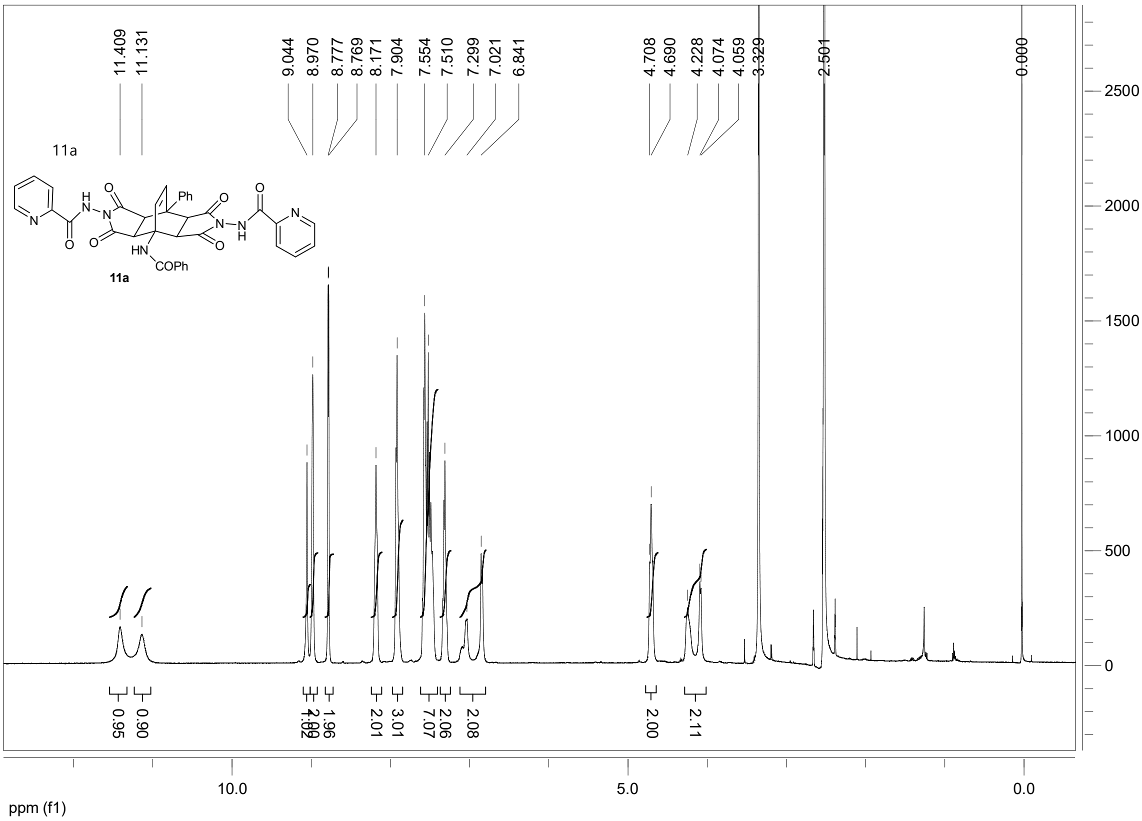

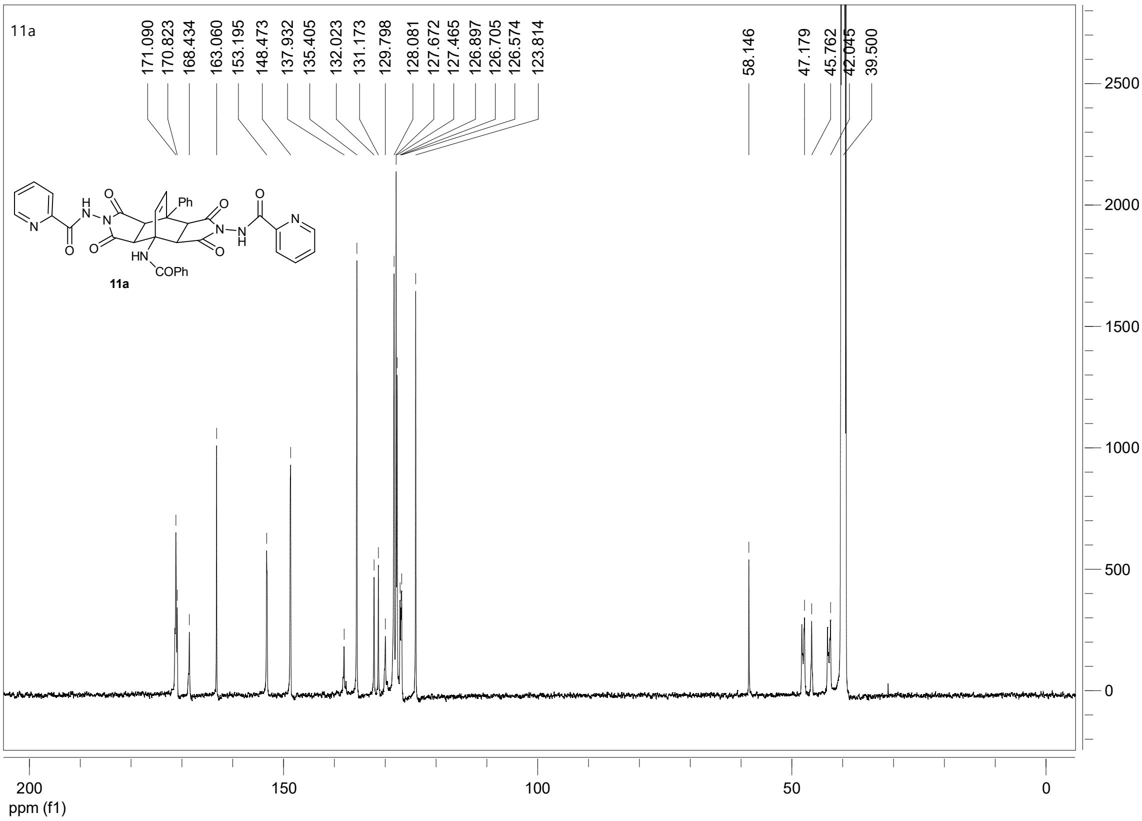

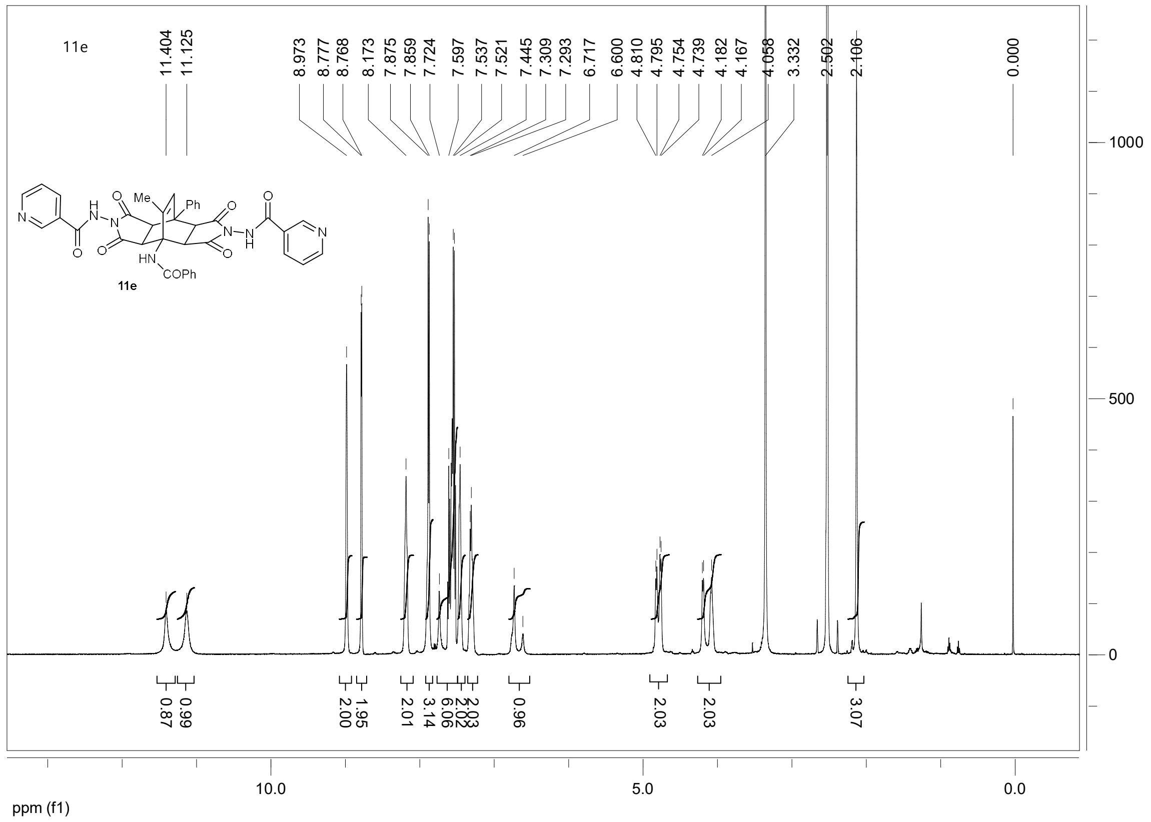
11e
11.404
11.125
8.973
8.777
8.768
8.173
7.875
7.859
7.724
7.597
7.537
7.521
7.445
7.309
7.293
6.717
6.600
4.810
4.795
4.754
4.739
4.182
4.167
4.058
3.332
2.502
2.106
0.000
Me
Ph
N
HN
COPh
11e
0.87
0.99
2.00
1.95
2.01
3.14
6.06
2.02
2.03
0.96
2.03
2.03
3.07
10.0
5.0
0.0
ppm (f1)
1000
500
0

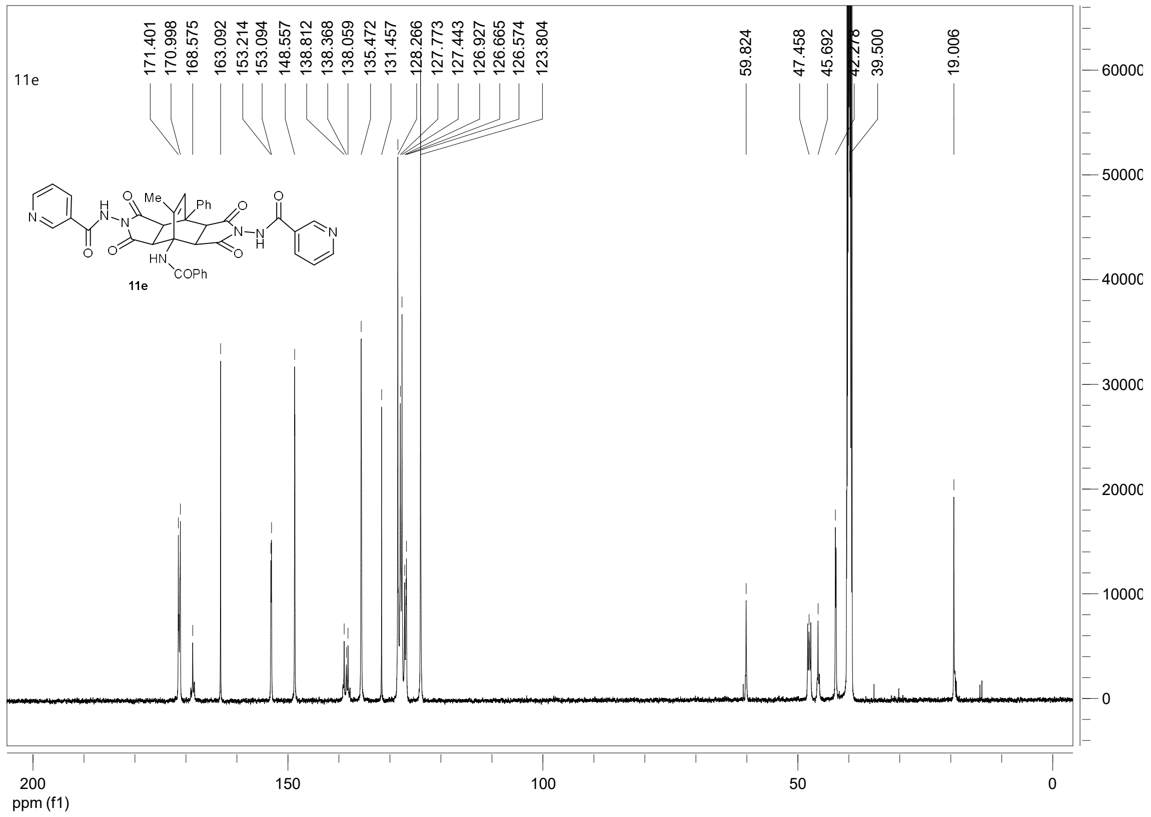
11e
171.401
170.998
168.575
163.092
153.214
153.094
148.557
138.812
138.368
138.059
135.472
131.457
128.266
127.773
127.443
126.927
126.665
126.574
123.804
59.824
47.458
45.692
42.276
39.500
19.006
Me
Ph
HN
COPh
11e
200
150
100
50
0
ppm (f1)
60000
50000
40000
30000
20000
10000
0
